# Reconstruction algorithms and arm positioning effects on abdominal CT image quality and radiation dose: a phantom study

**DOI:** 10.1186/s41747-026-00722-1

**Published:** 2026-05-07

**Authors:** Han Song Mun, Sanghyeok Lim, Shinhyung Kang

**Affiliations:** 1 Department of Radiology, Seoul St. Mary’s Hospital, College of Medicine, The Catholic University of Korea, Seoul, Republic of Korea; 2 Department of Radiology, Soonchunhyang University Hospital Bucheon, Soonchunhyang University College of Medicine, Bucheon-si, Gyeonggi-do, Republic of Korea; 3 GE Healthcare Co., Ltd, Seoul, Republic of Korea

**Keywords:** Image reconstruction, Multidetector computed tomography, Patient positioning, Phantoms (imaging), Radiation dosage

## Abstract

**Objective:**

To evaluate the effects of arm positioning and reconstruction algorithms on radiation dose and image quality of abdominal CT.

**Materials and methods:**

Abdominal CT scans were performed using a customized body phantom at 100 kVp with automatic tube current modulation based on noise indices (NI) of 9, 11, and 13 across seven arm positions: arms up (AU), arms down (AD), arms down with one or two-layered air cushions (ADAC, ADAC2), arms on the belly (AB), and arms on the belly with one or two-layered air cushions (ABAC, ABAC2). Images were reconstructed using filtered back projection (FBP), iterative reconstruction, and deep learning-based reconstruction (DLIR). Radiation dose was recorded. Quantitative image quality metrics included image noise, signal-to-noise ratio (SNR), contrast-to-noise ratio (CNR), sharpness, and structural similarity. Qualitative assessment of sharpness, noise, artifacts, and overall image quality was performed using fixed-dose AU-FBP images as the reference standard.

**Results:**

Compared with the AU position, radiation dose increased by up to 83% in non-AU configurations, with similar trends across all NI settings. In contrast, air cushion use caused only minor, configuration-dependent dose changes. Qualitatively, non-AU FBP images showed marked degradation, whereas DLIR enabled selected non-AU configurations to approach near-reference diagnostic quality. Air cushion-related benefits were modest overall but more apparent in AB than AD configurations.

**Conclusion:**

Arm positioning is the primary determinant of radiation dose and image quality in abdominal CT. When arm elevation is not feasible, DLIR effectively mitigates image quality degradation, while air cushions provide limited, configuration-dependent benefits.

**Relevance statement:**

When arms cannot be raised during abdominal CT, avoiding arms-on-belly and using air cushions may help maintain image quality without substantially increasing radiation dose, while deep learning image reconstruction further enhances diagnostic performance under restricted positioning conditions.

**Key Points:**

- Arm positioning is a major determinant of radiation dose and image quality in abdominal CT.
- Arms-on-belly positioning produces poorer image quality despite increased radiation dose.
- DLIR improves image quality across arm positions but does not eliminate positioning-related degradation.

**Graphical Abstract:**

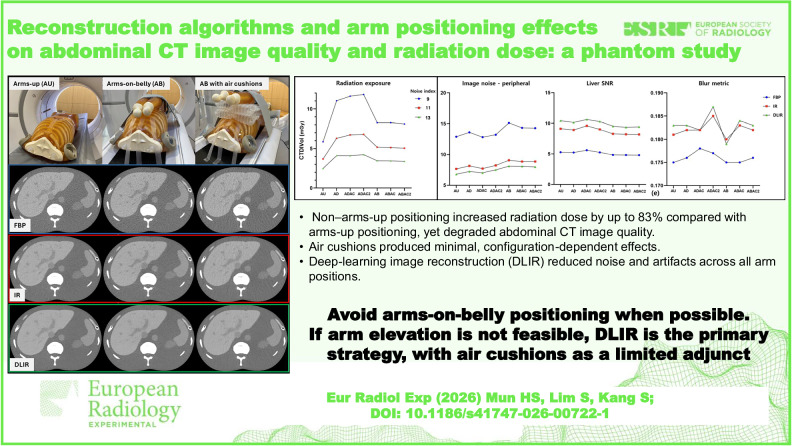

## Background

Computed tomography (CT) of the abdomen is usually performed with the patient’s arms positioned above their shoulders. However, some patients cannot raise their arms to this position because of trauma, altered mental status, or neurological disease. When the arms are lowered during a scan, beam-hardening artifacts and photon starvation occur, resulting in suboptimal image quality despite increased radiation exposure.

Previous studies have explored methods to optimize image quality in patients unable to elevate their arms. Iterative reconstruction (IR) algorithms have been shown to reduce beam-hardening artifacts in low-dose CT, while partial arm elevation or alternative arm positioning has also been proposed to improve image quality [1–3]. In addition to arm positioning, physical separation between the arms and torso using the air cushions has been suggested as a strategy to mitigate scatter and beam-hardening effects by reducing geometric coupling between osseous structures and the primary beam [4–6].

Automatic exposure control (AEC) systems modulate radiation dose based on photon attenuation to maintain diagnostic image quality [7–12], but they remain susceptible to increased noise and artifacts in non-arms-up configurations [13, 14]. Recently, deep learning image reconstruction (DLIR) techniques have demonstrated improved noise suppression and artifact reduction compared with conventional reconstruction methods [15]. However, because noise magnitude alone does not fully capture image quality, an integrated evaluation of arm positioning, AEC behavior, and reconstruction algorithms using both quantitative and qualitative metrics is required [16–18].

Accordingly, this study aimed to investigate the effects of arm positioning on radiation exposure and abdominal CT image quality under AEC conditions using a phantom model, and to evaluate whether air cushions and advanced reconstruction algorithms can mitigate image degradation associated with non-arms-up positioning.

## Methods

This study was conducted using an anthropomorphic phantom rather than a human patient; therefore, institutional review board approval was not required.

### Phantom and arm positioning

A whole-body phantom (PBU-60; Kyoto Kagaku Co., Ltd.) simulating a man 165 cm tall and weighing 50 kg was used for this study. The phantom was comprised of a radiolucent soft tissue substitute embedded with a life-sized synthetic skeleton and organs, including the liver, kidneys, spleen, pancreas, stomach, and intestines. The phantom was placed on the CT table feet-first in a supine position in the center of the gantry.

**Fig. 1:**
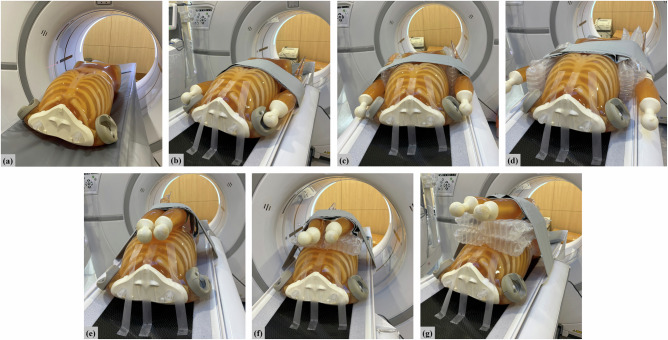
Photograph of a phantom in various arm positions, with and without air cushions, used for performing abdominal CT scans. The phantom is placed on a CT table in the following positions: (**a**) arms up AU; (**b**) arms down alongside the torso (AD); (**c**) AD with a single layer of air cushions (ADAC); (**d**) AD with double-layered air cushions (ADAC2); (**e**) arms placed atop the belly (AB); (**f**) AB with a single layer of air cushions (ABAC); and (**g**) AB with double layered air cushions (ABAC2)

Abdominal CT scans were obtained in the following seven arm positions: (1) standard arm-up position (AU), (2) arms down alongside the torso (AD), (3) arms down alongside the torso with a single layer of air cushions placed between the torso and each arm (ADAC), (4) arms down alongside the torso with double-layered air cushions placed between the torso and each arm (ADAC2), (5) arms placed atop the belly (AB), (6) arms placed atop the belly with a single layer of air cushions between the belly and each arm (ABAC), and (7) arms placed atop the belly with double-layered air cushions between the belly and each arm (ABAC2) (Fig. 1).

The air cushions were polyethylene–polyamide composite inflatable air-column cushions, measuring 40 × 24 cm before inflation and approximately 34 × 16 × 8 cm after full inflation. The estimated internal air pressure was 0.07–0.1 bar according to the manufacturer’s specifications. To improve placement reproducibility, each cushion was fully inflated before scanning and positioned at predefined contact points along the lateral chest wall or upper abdomen, maintaining a consistent separation. In the air-cushion configurations, predefined separations were maintained: 2.3 cm (ABAC) and 5.0 cm (ABAC2) between the arms and abdomen, and 3.2 cm (ADAC) and 6.5 cm (ADAC2) between the arms and torso. All distances were measured at the level of minimal separation between the air cushion and the torso and were kept constant across scans.

### CT acquisition and image reconstruction algorithms

CT imaging was performed on a 256-channel multi-detector CT system (Revolution CT; GE Healthcare) using the following parameters: detector configuration, 0.625 × 128 mm; gantry rotation speed, 0.6 s; pitch, 0.992; detector coverage, 80 mm; field of view, 350 mm; slice thickness, 2.5 mm; and slice interval, 2.5 mm. The CT scans were obtained using a fixed tube voltage of 100 kVp, while the tube current was automatically determined by the AEC system (Smart-mA; GE Healthcare) without predefined upper or lower limits based on the scout image and the preset noise index (NI). The NI levels were set to 9, 11, and 13, and five repeated scans were performed at each NI level. The scan range extended from the base of the lungs through the entire liver, with a standardized scan length of approximately 25 cm across all acquisitions.

The images were reconstructed using FBP, IR (50% ASIR-V®; GE Healthcare), and DLIR (medium strength level, TrueFidelity™; GE Healthcare). All reconstructed images were transferred to a workstation (Advantage Workstation 4.7; GE Healthcare). Further analysis was performed on MATLAB version 9.9 (R2020b; The MathWorks Inc.).

### Radiation dose measurements

Radiation dose descriptors, including the CT dose index volume (CTDIvol), which was measured in milligrays (mGy), were recorded after the completion of each CT examination for all image datasets. The CTDIvol and dose-length product were extracted from the dose report generated by the scanner for each scan. Each scanning condition was repeated five times, and the mean and standard deviation of CTDIvol and DLP across the five repeated acquisitions were used for analysis.

### Quantitative analysis for objective image quality assessment

A physician (S.K., with 11 years of experience in biomedical engineering and medical image processing/analysis) performed quantitative assessments of image noise, signal-to-noise ratio (SNR), contrast-to-noise ratio (CNR), image sharpness, and overall image quality. For quantitative measurements, circular regions of interest (ROIs) with a uniform area of approximately 50 mm² were used. The ROIs were copied and pasted across all image series to ensure that measurements were obtained at the same anatomical level, location, and size (Supplementary Fig. S1). All quantitative parameters were derived from five repeated acquisitions under identical scanning conditions, and the mean and standard deviation were calculated.

Image noise was assessed using three consecutive axial images. Central and peripheral image noise were evaluated separately and defined as the mean standard deviation of CT attenuation across the ROIs. Central image noise was measured using nine circular ROIs (three ROIs per slice) placed in the fat immediately anterior to the aorta. Peripheral image noise was measured using thirty-six circular ROIs placed in the subcutaneous fat layer, including the anterior abdominal wall (six ROIs per slice) and the left and right abdominal walls (three ROIs per side per slice).

To calculate SNR and CNR of the liver, three consecutive axial images were analyzed, with six ROIs placed in the liver on each slice. SNR and CNR for the liver were calculated using the following formulas:

$${{{\rm{SNR}}}}={{{\rm{Organ}}}}\; {{{\rm{HU}}}}/{{{\rm{Organ}}}}\; {{{\rm{standard}}}}\; {{{\rm{deviation}}}}$$

$${{{\rm{CNR}}}}=({{{\rm{Organ\; HU}}}}-{{{\rm{Vessel\; HU}}}})/{{{\rm{Image\; noise}}}}$$

Image sharpness was evaluated using no-reference blur metric algorithms, which assess sharpness based on adjacent pixel variation. A blur metric program was used to calculate the blur metric value for each image. Variations in intensity between adjacent pixels were first assessed in the original images, followed by low-pass-filtered versions of the images. The degree of blur was then determined by comparing the differences in intensity variations between the original and filtered images, with the resulting metric values expressed on a scale from 0 to 1, with lower values indicating sharper images and higher values indicating more blurred images [19].

The structural similarity (SSIM) index, which compares structural changes between an image and a reference image (using a full-reference method) by evaluating factors such as luminance, contrast, and structural (texture) information, was used to assess the overall image quality. The index was computed numerically, ranging from -1 (no similarity) to 1 (identical) [20–23]. The reference image for SSIM measurement was defined as the image reconstructed using fixed-dose FBP in the AU position (tube voltage, 100 kVp; tube current, 500 mA).

Since SSIM and blur values can vary depending on the position of the arms between images, a mask was created to define a specific region of interest (ROI), allowing us to quantitatively measure the potential impact of arm interference (Supplementary Fig. S2).

Quantitative analyses presented in the main manuscript were based exclusively on images acquired at a noise index of 11, while analyses at other noise index settings are provided in the Supplementary Material.

### Qualitative analysis for subjective image quality assessment

Two board-certified radiologists (H.S.M. and S.L., with 13 and 15 years of experience, respectively) independently reviewed the CT images at their respective hospitals using two different picture archiving and communication systems (ZeTTA PACS from Taeyoung Electronics; Deja-View version 3.0 from DongEun IT). The reviewers initially scored each imaging parameter using a five-point grading scale, modified from previously published image quality assessment schemes [24], with fixed-dose CT images (tube voltage, 100 kVp; tube current, 500 mA) acquired in the AU position and reconstructed with filtered back projection (FBP) serving as the reference standard. All study images were evaluated side-by-side with the corresponding reference images.

The specified criteria included sharpness, noise, artifacts, and overall image quality (Table 1). The reviewers assessed the quality of other image sets obtained using different reconstruction algorithms and arm positions relative to the reference image. All datasets were consistently displayed with soft-tissue window settings (window/level, 400/40 HU). For sharpness, noise, and overall image quality, a score of 3 was assigned when image quality was comparable to that of the reference image. For artifacts, given the near absence of artifacts in the reference image, images with a comparable artifact level were assigned a score of 4. Interobserver agreement for subjective image quality evaluation was assessed, and the scores from the two reviewers were retained for subsequent statistical analysis.

**Table 1:** Grading scale for qualitative analysis of CT examinations

Scale	Image quality parameters			
	Sharpness	Noise	Artifacts	Overall image quality
1	Severely blurred	Severe noise	Prominent and interfering	Unacceptable
2	Suboptimal	Moderate noise	Mild but present	Suboptimal
3	Acceptable	Mild noise	Minimal or none	Acceptable
4	Better than average	Less than mild	Virtually none	Good
5	Excellent	Minimal	None	Excellent

### Statistical analysis

Continuous variables are presented as mean ± standard deviation. Normality was assessed using the Shapiro–Wilk test. Radiation dose parameters and quantitative image quality metrics, including image noise, SNR, CNR, blur metric, and SSIM, were compared across arm positions and reconstruction methods using two-way analysis of variance (ANOVA), followed by Bonferroni-corrected post hoc pairwise comparisons when appropriate. Although normality was violated in two arm position configurations (ADAC2 and ABAC2) for radiation dose parameters, two-way ANOVA was applied because of its robustness to moderate deviations from normality in balanced designs. For SSIM, which is a bounded metric and violates assumptions of normality and homogeneity of variance, an additional non-parametric factorial analysis using the aligned rank transform (ART) approach was performed as a sensitivity analysis. Interobserver agreement for qualitative image quality assessment was evaluated using the intraclass correlation coefficient (ICC), calculated with a two-way random-effects model for absolute agreement. Qualitative image quality scores were analyzed separately for each domain using the Friedman test based on five repeated image series per arm position and reconstruction method, with Dunn’s test and Bonferroni correction applied for post hoc comparisons.

All statistical analyses were performed using MedCalc Statistical Software (version 22.0; MedCalc Software Ltd.), OriginPro (version 10.1, 2024; OriginLab Corporation), and R software (version 4.5.2; R Foundation for Statistical Computing). All tests were two-sided, and a *p* value < 0.05 was considered statistically significant.

## Results

### Radiation dose

Radiation dose was lowest in the AU position (CTDIvol, 3.68 ± 0.02 mGy), and all other arm configurations were associated with significantly higher radiation dose compared with AU (all *p* < 0.001). All radiation dose comparisons and percentage differences were calculated using the AEC-controlled AU acquisition as the reference. The CTDIvol increased by 74% in the AD position (6.41 ± 0.10 mGy) and by 40% in the AB position (5.17 ± 0.04 mGy) relative to AU. All AD configurations (AD, ADAC, and ADAC2) demonstrated significantly higher radiation dose than all AB configurations (AB, ABAC, and ABAC2) (all *p* ≤ 0.001).

**Fig. 2:**
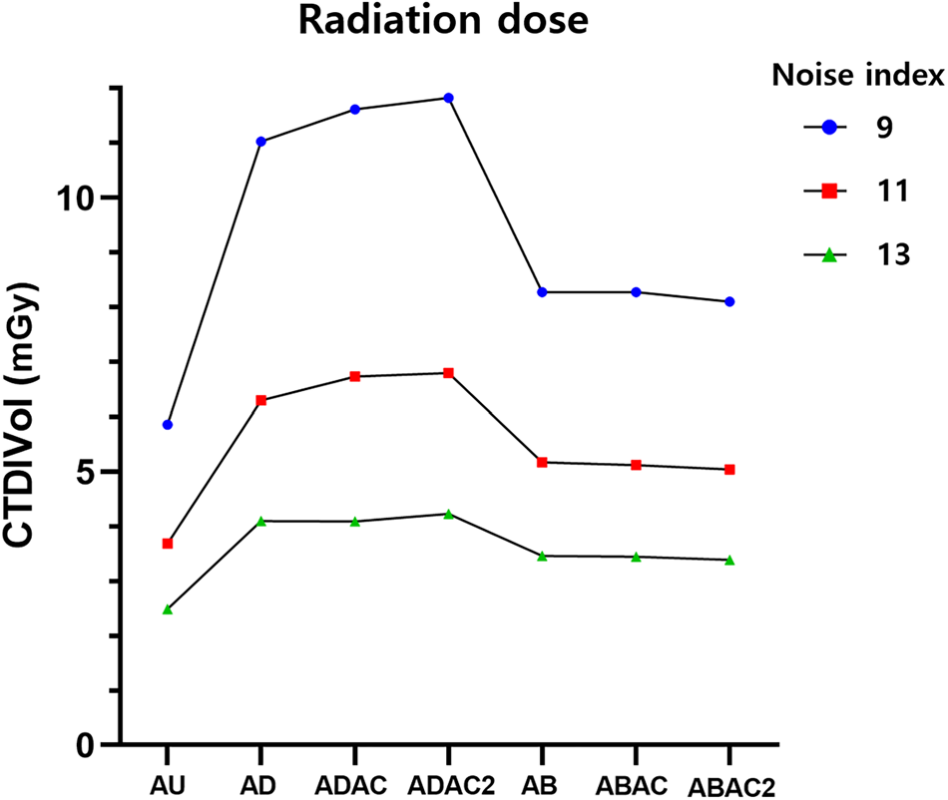
Radiation exposure (CTDIvol) according to noise index level and arm positions. AB, Arms placed atop the belly; ABAC, Arms placed atop the belly with a single layer of air cushions; ABAC2, Arms placed atop the belly with double-layered air cushions; AD, Arms down alongside the torso; ADAC, Arms down alongside the torso with a single layer air cushions; ADAC2, Arms down alongside the torso with double-layered air cushions; AU, Arms up; CTDIvol, Computed tomography dose index volume

Air-cushion insertion did not significantly reduce radiation dose in AD configurations, with no significant differences observed between AD and ADAC or between AD and ADAC2. In contrast, in AB configurations, air-cushion use was associated with a small but statistically significant reduction in radiation dose, with ABAC2 showing a lower CTDIvol than AB (*p* = 0.041) and ABAC (*p* = 0.030). Radiation dose at NI = 11 is summarized in Table 2, with trends across all evaluated noise index settings illustrated in Fig. 2 and detailed dose metrics provided in Supplementary Table S1.

**Table 2:** Radiation dose according to different arm positions during abdominal CT scans (noise index = 11)

	Tube voltage (kVp)	Tube current (range, mA)	CTDIvol (mGy)	DLP (mGy × cm)
AU	100	110–179	3.68 ± 0.02	114.35 ± 0.42
AD	100	165–295	6.41 ± 0.10	199.19 ± 3.07
ADAC	100	172–296	6.66 ± 0.08	206.88 ± 2.59
ADAC2	100	172–304	6.73 ± 0.12	209.02 ± 3.77
AB	100	161–235	5.17 ± 0.04	160.08 ± 0.51
ABAC	100	161–227	5.12 ± 0.02	158.90 ± 0.58
ABAC2	100	153–225	5.05 ± 0.00	156.79 ± 0.20
AU (reference standard)^†^	100	500	12.79	397.07

Data are presented as mean ± standard deviation unless otherwise specified

*AB* Arms placed atop the belly, *ABAC* Arms placed atop the belly with a single layer of air cushions, *ABAC2* Arms placed atop the belly with double-layered air cushions, *AD* Arms down alongside the torso, *ADAC* Arms down alongside the torso with a single layer of air cushions, *ADAC2* Arms down alongside the torso with double-layered air cushions, *AU* Arms up; *CTDIvol* Volume CT dose index, *DLP* Dose-length product

^†^ The fixed-dose AU acquisition (100 kVp, 500 mA; CTDIvol = 12.79 mGy) was used exclusively as a reference image for qualitative assessment and SSIM analysis, and was not included in any radiation dose comparisons. All dose comparisons and percentage differences reported in this study refer to the AEC-controlled AU acquisition (CTDIvol = 3.68 ± 0.02 mGy)

### Objective image quality

**Fig. 3:**
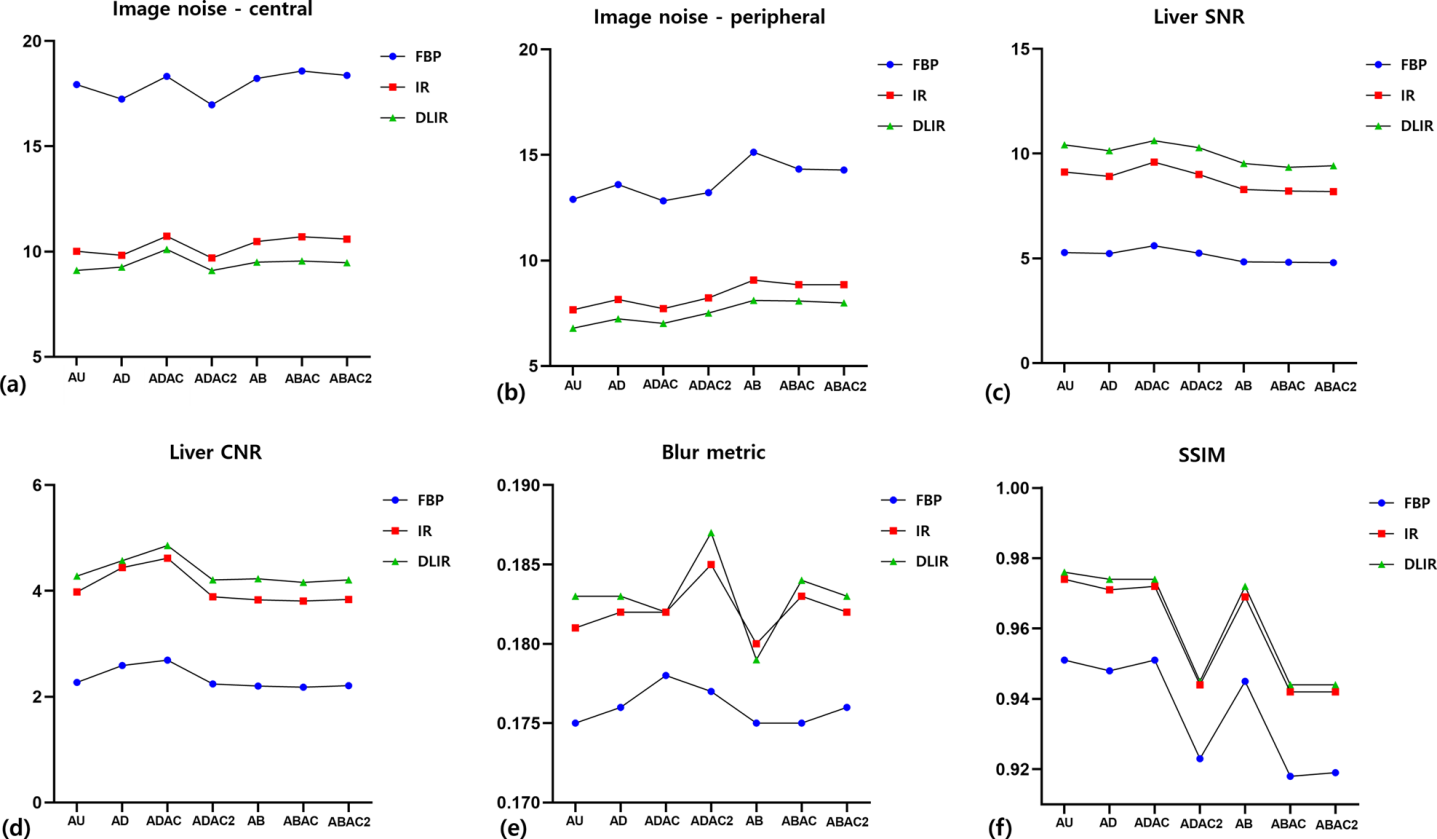
Central (**a**) and peripheral (**b**) image noise as influenced by arm position, the presence of an air cushion, and reconstruction algorithms. Liver SNR (**c**) and CNR (**d**) under the same conditions. **e** Blur metric reflecting image sharpness, calculated based on adjacent pixel variation. **f** Overall image quality assessed using SSIM. AB, Arms placed atop the belly; ABAC, Arms placed atop the belly with a single layer of air cushion; ABAC2, Arms placed atop the belly with double-layered air cushions; AD, Arms down alongside the torso; ADAC, Arms down alongside the torso with a single layer of air cushion; ADAC2, Arms down alongside the torso with double-layered air cushions; AU, Arms up; CNR, Contrast-to-noise ratio; DLIR, Deep learning image reconstruction; FBP, Filtered back projection; IR, Iterative reconstruction; SNR, Signal-to-noise ratio; SSIM, Structural similarity index

Quantitative image quality results, including image noise, liver SNR and CNR, blur metric, and SSIM, are summarized in Table 3 and Fig. 3. Corresponding analyses across different noise index settings are provided in Supplementary Table S2 and Supplementary Fig. S3.

**Table 3:** Quantitative image quality metrics based on arm positions and reconstruction algorithms (noise index = 11)

Arm position	Reconstruction algorithm	Central noise	Peripheral noise	Liver SNR	Liver CNR	Blur metric	SSIM
AU (reference)	FBP	11.01 ± 1.78	7.43 ± 1.31	9.11 ± 1.72	4.72 ± 0.91	0.184 ± 0.003	1.000
AU	FBP	17.94 ± 2.54	12.9 ± 2.2	5.28 ± 0.76	2.27 ± 0.55	0.175 ± 0.003	0.951 ± 0.002
	IR	10.01 ± 1.65	7.66 ± 1.49	9.12 ± 1.51	3.98 ± 0.99	0.181 ± 0.004	0.974 ± 0.001
	DLIR	9.11 ± 1.45	6.79 ± 1.27	10.42 ± 1.49	4.28 ± 1.04	0.183 ± 0.004	0.976 ± 0.001
AD	FBP	17.25 ± 2.76	13.6 ± 2.68	5.23 ± 0.79	2.59 ± 0.59	0.176 ± 0.003	0.948 ± 0.002
	IR	9.82 ± 1.89	8.15 ± 1.8	8.92 ± 1.57	4.44 ± 0.91	0.182 ± 0.003	0.971 ± 0.001
	DLIR	9.26 ± 1.75	7.24 ± 1.39	10.14 ± 1.67	4.57 ± 0.9	0.183 ± 0.004	0.974 ± 0.001
ADAC	FBP	18.33 ± 3.23	12.83 ± 2.45	5.6 ± 1.07	2.69 ± 0.41	0.178 ± 0.002	0.951 ± 0.002
	IR	10.73 ± 2.16	7.72 ± 1.64	9.6 ± 2.04	4.62 ± 0.92	0.182 ± 0.003	0.972 ± 0.001
	DLIR	10.1 ± 1.97	7.02 ± 1.33	10.62 ± 2.07	4.86 ± 0.89	0.182 ± 0.004	0.974 ± 0.001
ADAC2	FBP	16.97 ± 3.47	13.22 ± 3.6	5.25 ± 1.12	2.24 ± 0.37	0.177 ± 0.003	0.923 ± 0.005
	IR	9.7 ± 2.31	8.23 ± 3.66	9.01 ± 2.16	3.89 ± 0.51	0.185 ± 0.004	0.944 ± 0.005
	DLIR	9.1 ± 2.12	7.51 ± 3.45	10.29 ± 2.18	4.21 ± 0.58	0.187 ± 0.003	0.945 ± 0.005
AB	FBP	18.23 ± 3.03	15.13 ± 2.83	4.83 ± 0.81	2.2 ± 0.64	0.175 ± 0.003	0.945 ± 0.001
	IR	10.48 ± 1.93	9.07 ± 1.96	8.29 ± 1.68	3.83 ± 1.32	0.18 ± 0.002	0.969 ± 0.001
	DLIR	9.5 ± 1.71	8.11 ± 1.54	9.53 ± 1.87	4.23 ± 1.27	0.179 ± 0.003	0.972 ± 0.001
ABAC	FBP	18.58 ± 3.03	14.33 ± 3.32	4.81 ± 0.75	2.18 ± 0.55	0.175 ± 0.002	0.918 ± 0.005
	IR	10.7 ± 2.24	8.85 ± 3.19	8.22 ± 1.52	3.81 ± 1.04	0.183 ± 0.003	0.942 ± 0.005
	DLIR	9.55 ± 1.81	8.08 ± 2.89	9.35 ± 1.62	4.16 ± 1.08	0.184 ± 0.003	0.944 ± 0.005
ABAC2	FBP	18.37 ± 2.99	14.29 ± 3.08	4.8 ± 0.89	2.21 ± 0.34	0.176 ± 0.002	0.919 ± 0.005
	IR	10.59 ± 2.13	8.85 ± 3.11	8.19 ± 1.69	3.84 ± 0.61	0.182 ± 0.003	0.942 ± 0.004
	DLIR	9.47 ± 1.9	7.99 ± 2.94	9.42 ± 1.75	4.21 ± 0.71	0.183 ± 0.003	0.944 ± 0.005

Data are presented as mean ± standard deviation

*AB* Arms placed atop the belly, *ABAC* Arms placed atop the belly with a single layer of air cushions, *ABAC2* Arms placed atop the belly with double-layered air cushions, *AD* Arms down alongside the torso, *ADAC* Arms down alongside the torso with a single layer of air cushions, *ADAC2* Arms down alongside the torso with double-layered air cushions, *AU* Arms up, *CNR* Contrast-to-noise ratio, *DLIR* Deep learning image reconstruction, *FBP* Filtered back projection, *IR* Iterative reconstruction, *SNR* Signal-to-noise ratio, *SSIM* Structural similarity index

### Image noise

Arm position and reconstruction algorithm had significant main effects on both central and peripheral image noise (all *p* < 0.001), with no significant interaction between the two factors. Among reconstruction algorithms, FBP consistently yielded the highest noise, followed by IR and DLIR (all *p* < 0.001).

For central image noise, differences according to arm position were statistically significant but modest compared with the effect of reconstruction. In contrast, peripheral noise showed a stronger dependence on arm positioning. Specifically, peripheral noise was significantly higher in AB compared with AU (mean difference = 1.65, *p* < 0.001) and ADAC (mean difference = 1.58, *p* < 0.001). Similarly, ABAC and ABAC2 demonstrated significantly higher peripheral noise than AU (mean differences = 1.30 and 1.26, respectively; both *p* < 0.001). In contrast, no significant difference in peripheral noise was observed between ADAC and AU, indicating mitigation of peripheral noise amplification by arm–body separation (Fig. 3a, b).

### Liver SNR and CNR

Liver SNR differed significantly according to arm position (*p* < 0.001), with AB configurations showing lower SNR than AD configurations and the AU position (all adjusted *p* ≤ 0.001). Among AD configurations, ADAC demonstrated significantly higher liver SNR than AD (*p* = 0.003) and ADAC2 (*p* = 0.030), whereas no significant difference was observed between AU and any AD configuration (all *p* ≥ 0.227). Liver SNR also differed significantly across reconstruction algorithms (*p* < 0.001), with DLIR yielding the highest values, followed by IR and FBP.

Liver CNR showed similar but less pronounced trends. ADAC demonstrated significantly higher liver CNR than AB and ABAC configurations (all *p* ≤ 0.002) and also higher CNR than AU (*p* = 0.014). In contrast, the use of two air cushions resulted in significantly lower liver CNR compared with the single-cushion configuration (*p* = 0.003). Across reconstruction algorithms, liver CNR was highest with DLIR, followed by IR and FBP (all *p* ≤ 0.012) (Fig. 3c, d).

### Blur metric

Two-way analysis of variance demonstrated significant main effects of arm position (*F* = 11.48, *p* < 0.001) and reconstruction algorithm (*F* = 169.17, *p* < 0.001) on the blur metric, as well as a significant interaction effect (*F* = 1.88, *p* = 0.037). Overall, AB configurations demonstrated lower blur values, whereas the ADAC2 configuration consistently showed the highest degree of image blur. Notably, AB exhibited significantly lower blur values than ADAC2 (*p* < 0.001), and differences across arm positions were more pronounced with IR and DLIR than with FBP (Fig. 3e).

For reference, the blur metric of full-dose AU-FBP images was 0.184 ± 0.003, showing minimal variability and serving as a stable reference for structural sharpness. Although image noise was consistently reduced with advanced reconstruction, blur metric values increased in selected configurations, suggesting a potential trade-off between noise suppression and structural sharpness.

### SSIM

SSIM differed significantly according to both arm position and reconstruction method (both *p* < 0.001). AU and AD configurations demonstrated significantly higher SSIM values than ABAC and ABAC2 (all *p* < 0.001), while ADAC maintained SSIM values comparable to AU. Across reconstruction methods, IR and DLIR consistently yielded higher SSIM values than FBP (*p* < 0.001). Although a modest interaction between arm position and reconstruction method was observed (*p* = 0.033), the overall pattern of SSIM variation remained consistent across arm positions and reconstruction techniques (Fig. 3f).

### Subjective image quality

**Fig. 4:**
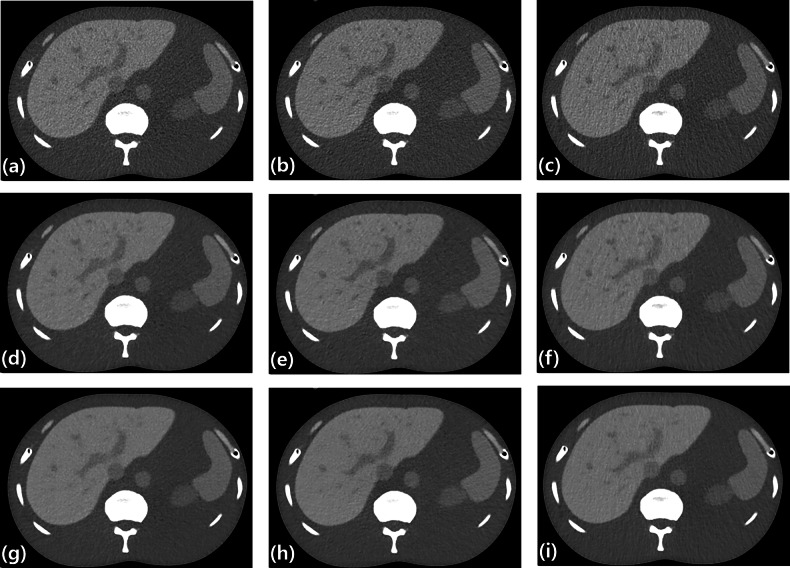
Representative axial abdominal CT images at the same slice level illustrating the effects of arm position and reconstruction algorithm on image quality. **a**–**c** Show images reconstructed with FBP for AU (**a**), AB (**b**), and ABAC2 (**c**). **d**–**f** Show the corresponding images reconstructed with IR for AU (**d**), AB (**e**), and ABAC2 (**f**). **g**–**i** Show images reconstructed with DLIR for AU (**g**), AB (**h**), and ABAC2 (**i**). All images were acquired at 100 kVp with AEC (noise index = 11) and displayed using soft-tissue window settings (window/level = 400/40 HU). AB, Arms placed atop the belly; ABAC2, Arms placed atop the belly with double-layered air cushions; AU, Arms up; DLIR, Deep learning image reconstruction; FBP, Filtered back projection; IR, Iterative reconstruction

Qualitative image quality scores differed significantly among arm positions and reconstruction methods across all evaluated domains (sharpness, noise, artifacts, and overall image quality; all *p* < 0.001), with high interobserver agreement (ICC = 0.839; 95% CI, 0.818–0.857; *p* < 0.001). For overall image quality, non-AU FBP images showed significantly lower scores compared with the reference images (AU-FBP, 2.0 ± 0.0 *versus* AB-FBP, 1.5 ± 0.0). In contrast, DLIR enabled selected non-AU configurations—particularly those using air cushions—to achieve near-reference overall image quality (ADAC2-DLIR and ABAC2-DLIR, both 3.0 ± 0.0). Representative qualitative differences in image quality according to arm position and reconstruction algorithm are illustrated in Fig. 4 and Supplementary Fig. S4. A comprehensive summary of qualitative image quality scores stratified by noise index is provided in Table 4 and Supplementary Table S3.

**Table 4:** Qualitative analysis of CT images based on arm positions and reconstruction algorithms (noise index = 11)

Reconstruction algorithm	Arm position	Sharpness	Noise	Artifacts	Overall Image Quality
FBP AU (reference standard)		3	3	4	3
FBP	AU	2.4 ± 0.4	2.0 ± 0.0	4.0 ± 0.0	2.0 ± 0.0
	AD	2.0 ± 0.0	1.5 ± 0.4	1.8 ± 0.3	2.0 ± 0.0
	ADAC	3.0 ± 0.0	1.8 ± 0.3	2.1 ± 0.2	2.0 ± 0.0
	ADAC2	3.0 ± 0.0	1.4 ± 0.2	2.5 ± 0.0	2.2 ± 0.3
	AB	2.0 ± 0.0	2.0 ± 0.0	1.8 ± 0.3	1.5 ± 0.0
	ABAC	2.1 ± 0.2	1.5 ± 0.0	1.5 ± 0.0	2.0 ± 0.0
	ABAC2	2.7 ± 0.3	2.0 ± 0.0	2.0 ± 0.0	2.0 ± 0.0
IR	AU	2.1 ± 0.2	2.7 ± 0.3	4.0 ± 0.0	2.8 ± 0.3
	AD	2.0 ± 0.0	1.9 ± 0.2	2.1 ± 0.2	2.3 ± 0.3
	ADAC	2.8 ± 0.3	2.2 ± 0.4	2.3 ± 0.3	2.2 ± 0.3
	ADAC2	2.9 ± 0.2	2.3 ± 0.3	2.3 ± 0.4	2.7 ± 0.3
	AB	2.5 ± 0.0	2.5 ± 0.0	2.0 ± 0.0	2.0 ± 0.0
	ABAC	2.7 ± 0.3	2.2 ± 0.3	2.1 ± 0.2	2.3 ± 0.3
	ABAC2	2.8 ± 0.3	2.4 ± 0.2	2.3 ± 0.3	2.3 ± 0.3
DLIR	AU	2.4 ± 0.2	3.0 ± 0.0	4.0 ± 0.0	3.0 ± 0.0
	AD	2.0 ± 0.0	2.0 ± 0.0	2.5 ± 0.4	2.8 ± 0.3
	ADAC	2.6 ± 0.2	2.5 ± 0.0	2.6 ± 0.2	2.8 ± 0.3
	ADAC2	2.8 ± 0.3	2.9 ± 0.2	2.8 ± 0.3	3.0 ± 0.0
	AB	2.0 ± 0.0	2.5 ± 0.0	3.0 ± 0.0	2.0 ± 0.0
	ABAC	2.3 ± 0.3	3.0 ± 0.0	3.0 ± 0.0	2.2 ± 0.3
	ABAC2	2.5 ± 0.0	3.0 ± 0.0	3.0 ± 0.0	3.0 ± 0.0

Data are presented as mean ± standard deviation unless otherwise specified. Interobserver agreement for the subjective image quality evaluation was high (intraclass correlation coefficient, 0.839; 95% confidence interval, 0.818–0.857; *p* < 0.001)

*AB* Arms placed atop the belly, *ABAC* Arms placed atop the belly with a single layer air cushions, *ABAC2* Arms placed atop the belly with double-layered air cushions, *AD* Arms down alongside the torso, *ADAC* Arms down alongside the torso with a single layer air cushions, *ADAC2* Arms down alongside the torso with double-layered air cushions, *AU* Arms up, *DLIR* Deep learning image reconstruction, *FBP* Filtered back projection, *IR* Iterative reconstruction

## Discussion

The present study was motivated by a practical clinical question: identifying the most reasonable imaging strategy when AU positioning cannot be achieved, rather than reaffirming its superiority. By systematically evaluating arm positioning, reconstruction algorithms, and the use of air cushions under realistic AEC conditions, and by integrating quantitative and qualitative image quality metrics, this study provides a comprehensive framework for understanding how these factors jointly influence abdominal CT image quality.

Previous studies have primarily evaluated CT image quality by manipulating tube voltage, tube current, or reconstruction algorithms [1, 15, 25, 26], generally reporting improved image quality with higher radiation exposure or comparable quality with advanced reconstruction at reduced dose. In contrast, the present study fixed tube voltage and noise index under AEC conditions, thereby more closely reflecting routine clinical practice and allowing the isolated assessment of arm positioning effects.

The finding that AD configurations resulted in higher radiation dose than AB configurations, despite demonstrating better image quality, may appear counterintuitive at first glance. This can be explained by the behavior of AEC, which primarily responds to global attenuation and patient geometry rather than image degradation itself. In the AD position, the presence of the upper extremities alongside the torso increases the effective lateral attenuation over a broad angular range, leading the AEC system to increase tube output to maintain the target noise index, even though projection continuity and image quality are preserved.

Using the AU position as the reference at an NI of 11, radiation exposure increased substantially in all non-AU configurations, with a 74% increase in the AD position and a 40% increase in the AB position. Although air-cushion use resulted in small, configuration-dependent dose changes, no consistent or clinically meaningful dose reduction was observed. These findings align with prior reports demonstrating increased radiation exposure when the arms are positioned within the scan field during abdominal CT examinations [3, 14, 27, 28]. From a clinical perspective, the observed absolute dose differences, on the order of a few mGy, are modest and should be interpreted in the context of real-world practice. Patients who are unable to raise their arms are more often examined under specific clinical circumstances rather than typical imaging scenarios, and in this context, a small absolute increase in radiation dose should be considered alongside the need to maintain adequate diagnostic image quality.

Quantitative image quality analysis at NI = 11 demonstrated that arm positioning was a primary determinant of image quality, with distinct effects on peripheral and central noise. Peripheral noise was predominantly governed by arm position, reflecting local geometric factors such as arm–torso superimposition near the body surface. Notably, peripheral noise in the ADAC configuration was comparable to that of the AU reference, whereas AB configurations consistently exhibited higher peripheral noise. In contrast, central noise showed only modest variation across arm positions and was more strongly influenced by reconstruction algorithms, indicating that global attenuation and system-level noise modulation play a dominant role in central image regions.

Liver SNR and CNR translated these noise-related findings into clinically relevant measures of parenchymal image quality. AB configurations were consistently associated with lower SNR and CNR, indicating compromised signal stability and tissue contrast. Among non-AU configurations, the ADAC position demonstrated favorable SNR and CNR values comparable to the AU reference, supporting its role as a practical alternative when arm elevation is not feasible. Although advanced reconstruction uniformly improved SNR and CNR across all arm positions, it did not selectively compensate for the physical degradation induced by unfavorable arm positioning.

The observed degradation of image quality in AB configurations can be explained by projection geometry and attenuation characteristics. Quantitative and spatial noise analyses demonstrated increased noise and artifact conspicuity, particularly in anterior regions, consistent with direct arm–abdomen overlap. From a projection perspective, placing both upper extremities over the abdomen introduces more abrupt view-to-view changes in attenuation compared with AD configurations, which provide smoother transitions across projection angles. The presence of bilateral upper extremities containing osseous structures further increases structural complexity in the central abdominal region, thereby exacerbating streak artifacts despite increased radiation exposure. Although scatter effects are unlikely to be the primary driver of image degradation in this setting, arms-on-belly positioning may introduce slightly increased structure-dependent local scatter, which could further contribute to reduced image uniformity.

Because the blur metric of full-dose FBP images showed minimal variability, the observed increase in blur with IR and DLIR can reasonably be attributed primarily to reconstruction-related smoothing rather than positional or noise-related factors. In this context, the blur metric and SSIM provided complementary insights beyond noise-based indices by characterizing structural image fidelity. Despite favorable noise and SNR/CNR profiles, configurations involving air cushions—particularly those with double-cushion placement—demonstrated increased blur and reduced SSIM. This finding suggests that non-uniform air gaps and complex arm–body interfaces may introduce spatially heterogeneous attenuation and partial-volume effects that are subsequently smoothed by reconstruction algorithms, underscoring that noise reduction does not necessarily equate to preserved structural fidelity [19–23].

It should be noted that the applied blur metric is a no-reference measure and does not localize the underlying sources of image blur. Accordingly, the observed differences in blur may reflect a combination of edge preservation and texture smoothing rather than a single physical mechanism. This limitation is particularly relevant for iterative and deep learning–based reconstruction techniques, in which texture suppression may contribute to higher blur metric values despite improved noise characteristics.

Overall image quality was highest in the AU configuration and consistently inferior in AB configurations, primarily due to increased noise and artifact conspicuity. AD configurations were more frequently rated as diagnostically acceptable alternatives, particularly when combined with advanced reconstruction. Importantly, qualitative evaluation highlighted the distinction between continuous physical metrics and threshold-based clinical acceptability, clarifying which configurations remain usable in practice.

These patterns were preserved across multiple noise index settings. Across NI = 9, 11, and 13, the relative effects of arm positioning, reconstruction algorithm, and air-cushion use on image quality remained consistent, despite NI-dependent differences in absolute image quality. Spatially resolved noise analysis further revealed anterior-dominant noise amplification in AB configurations, consistent with localized beam attenuation and scatter caused by direct arm–abdomen overlap. This finding provides a quantitative explanation for the persistently inferior subjective noise and artifact scores observed in these configurations and reinforces the geometric origin of image degradation.

When quantitative and qualitative findings were integrated, the role of air cushions emerged as conditional rather than universal. While quantitative metrics alone might suggest modest noise-related benefits, qualitative evaluation demonstrated that only selected air-cushion configurations achieved acceptable diagnostic quality, and that excessive or non-uniform arm separation could offset potential benefits. Accordingly, air cushions should be regarded as a situational adjunct rather than a substitute for optimal arm positioning.

This study has several limitations. First, although an anthropomorphic phantom was used to approximate human anatomy, phantom-based experiments cannot fully replicate clinical imaging conditions, including contrast-enhanced examinations, interindividual variability in patient body habitus, and system-specific dose–noise behavior. In addition, this study was performed using a single CT system with a limited range of noise index settings, and a dedicated dose–noise calibration experiment was not conducted. These factors may limit the generalizability of the observed dose and image quality relationships to other scanner platforms and clinical scenarios. Second, the clinical applicability of air cushions may be constrained by their availability, patient compliance, and variability in cushion placement in routine practice. Finally, quantitative metrics such as blur and SSIM may reflect global structural differences rather than focal parenchymal image quality, and therefore should be interpreted as complementary rather than definitive measures of diagnostic performance.

In conclusion, arm positioning is a primary determinant of image quality in abdominal CT, with AB configurations consistently associated with increased noise, degraded structural fidelity, and reduced diagnostic quality. When AU positioning is not feasible, AD positioning combined with advanced reconstruction algorithms represents the most practical alternative, while the use of air cushions should be considered cautiously as a conditional adjunct rather than a substitute for optimal arm positioning.

## Supplementary information

**Additional file 1: Table S1**. Radiation dose according to different arm positions during abdominal CT scans. **Table S2**. Comprehensive quantitative analysis results. **Table S3**. Qualitative analysis of CT images according to noise index, arm position, and reconstruction algorithm. **Figure S1**. Representative axial abdominal CT image illustrating the placement of circular regions of interest (ROIs) used for quantitative image analysis. **Figure S2**. Representative axial abdominal CT image illustrating the application of a predefined mask used to define a specific region of interest (ROI) for quantitative analysis of SSIM and blur metrics. **Figure S3**. Quantitative image quality metrics stratified by noise index settings (NI = 9, 11, and 13). Panels (a–f), (g–l), and (m–r) correspond to NI values of 9, 11, and 13, respectively. **Figure S4**. Representative axial abdominal CT images demonstrating qualitative differences in image quality according to arm position and reconstruction method.

## Data Availability

The datasets generated and/or analyzed during the current study are not publicly available but are available from the corresponding author on reasonable request.

## Abbreviations

AB: Arms placed atop the belly
ABAC: Arms placed atop the belly with a single layer air cushions between the belly and each arm
ABAC2: Arms placed atop the belly with double-layered air cushions between the belly and each arm
AD: Arms down alongside the torso
ADAC: Arms down alongside the torso with a single layer of air cushions placed between the torso and each arm
ADAC2: Arms down alongside the torso with double-layered air cushions placed between the torso and each arm
AEC: Automatic exposure control
AU: Standard arm-up position
CT: Computed tomography
CTDIvol: CT dose index volume
DLIR: Deep learning image reconstruction
FBP: Filtered back projection
IR: Iterative reconstruction
ROI: Region of interest
SNR: Signal-to-noise ratio
SSIM: Structural similarity
