# Correction: Reconstruction algorithms and arm positioning effects on abdominal CT image quality and radiation dose: a phantom study

**DOI:** 10.1186/s41747-026-00752-9

**Published:** 2026-06-16

**Authors:** Han Song Mun, Sanghyeok Lim, Shinhyung Kang

**Affiliations:** 1 Department of Radiology, Seoul St. Mary’s Hospital, College of Medicine, The Catholic University of Korea, Seoul, Republic of Korea; 2 Department of Radiology, Soonchunhyang University Hospital Bucheon, Soonchunhyang University College of Medicine, Bucheon-si, Republic of Korea; 3 GE Healthcare Co., Ltd., Seoul, Republic of Korea

**Correction to: European Radiology Experimental**

10.1186/s41747-026-00722-1, published online 07 May 2026

In the original publication of this article, the numbers ‘9’ and ‘13’ on the right side of Figure 2 and in the corresponding graph within the graphical abstract were inadvertently switched during figure preparation. In addition, an unintended arrow mark was inadvertently included in the graphical abstract image. The figure and graphical abstract have now been corrected in the updated version of the article. These corrections do not affect the results, interpretation, or conclusions of the study.

The correct Fig. 2 is:

**Figure:**
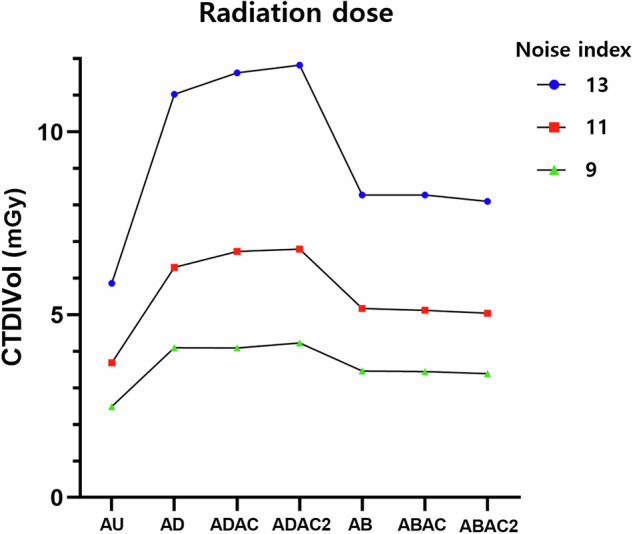

**Figure:**
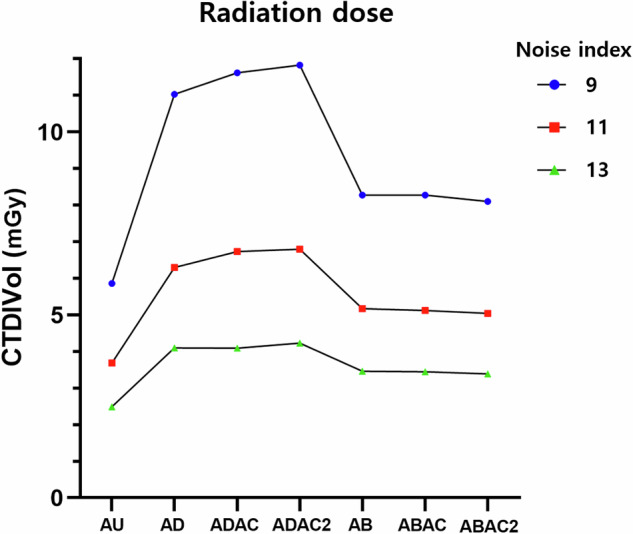

The correct graphical abstract is:

**Figure:**
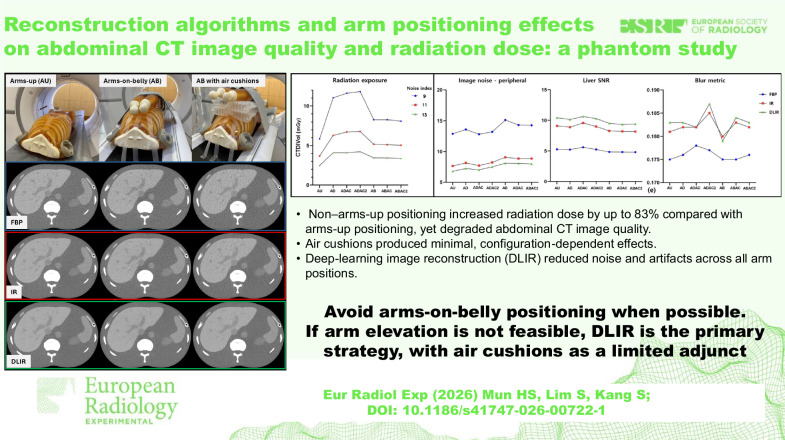

The original article has been corrected.

