# Supplementary material for: Reconstruction algorithms and arm positioning effects on abdominal CT image quality and radiation dose: a phantom study
Source: Eur Radiol Exp. 2026 May 7;10:61. doi: 10.1186/s41747-026-00722-1 (PMC13153281; doi:10.1186/s41747-026-00722-1)

Reconstruction algorithms and arm positioning effects on abdominal CT image quality and radiation dose: a phantom study

ELECTRONIC SUPPLEMENTARY MATERIAL

Supplementary Table S1. Radiation dose according to different arm positions during abdominal CT scans

Noise index	Arm position	Tube voltage (kVp)	Tube current (range, mA)	CTDIvol (mGy)	DLP (mGy*cm)
9	AU	100	172–292	5.88 ± 0.04	182.63 ± 1.07
	AD	100	281–519	11.29 ± 0.23	350.48 ± 7.31
	ADAC	100	288–521	11.61 ± 0.20	360.30 ± 6.40
	ADAC2	100	285–539	11.79 ± 0.19	366.19 ± 6.00
	AB	100	254–385	8.30 ± 0.03	257.76 ± 0.96
	ABAC	100	253–373	8.25 ± 0.03	256.15 ± 0.84
	ABAC2	100	242–367	8.12 ± 0.03	252.33 ± 0.81
11	AU	100	110–179	3.68 ± 0.02	114.35 ± 0.42
	AD	100	165–295	6.41 ± 0.10	199.19 ± 3.07
	ADAC	100	172–296	6.66 ± 0.08	206.88 ± 2.59
	ADAC2	100	172–304	6.73 ± 0.12	209.02 ± 3.77

	AB	100	161–235	5.17 ± 0.04	160.08 ± 0.51
	ABAC	100	161–227	5.12 ± 0.02	158.90 ± 0.58
	ABAC2	100	153–225	5.05 ± 0.00	156.79 ± 0.20
13	AU	100	76–119	2.49 ± 0.01	77.32 ± 0.37
	AD	100	109–183	4.06 ± 0.08	125.99 ± 2.60
	ADAC	100	114–184	4.13 ± 0.06	128.09 ± 1.85
	ADAC2	100	113–189	4.22 ± 0.07	130.91 ± 2.19
	AB	100	110–156	3.46 ± 0.01	107.30 ± 0.29
	ABAC	100	109–151	3.44 ± 0.01	106.82 ± 0.28
	ABAC2	100	105–149	3.39 ± 0.01	105.34 ± 0.19
AU (reference of standard)†		100	500	12.79	397.07

Data are presented as mean ± standard deviation unless otherwise specified. AB, arms placed atop the belly; ABAC, arms placed atop the belly with a single layer of air cushions; ABAC2, arms placed atop the belly with double-layered air cushions; AD, arms down alongside the torso; ADAC, arms down alongside the torso with a single layer of air cushions; ADAC2, arms down alongside the torso with double-layered air cushions; AU, arms up; CTDIvol, volume CT dose index; DLP, dose-length product.

†The fixed-dose AU acquisition (100 kVp, 500 mA; CTDIvol = 12.79 mGy) was used exclusively as a reference image for qualitative assessment and SSIM analysis, and was not included in any radiation dose comparisons.

Supplementary Table S2. Comprehensive quantitative analysis results

Noise index	Arm position	Reconstruction algorithm	Center noise	Anterior noise	Lateral noise	Peripheral noise	Liver SNR	Spleen SNR	Liver CNR	Spleen CNR	Blur metric	SSIM
Reference of standard	AU	FBP	11.01 ± 1.78	7.04 ± 0.98	7.81 ± 1.51	7.43 ± 1.31	9.11 ± 1.72	6.29 ± 0.92	4.72 ± 0.91	1.95 ± 0.6		
9	AU	FBP	14.21 ± 2.7	9.85 ± 1.8	10.59 ± 2.11	10.22 ± 1.99	6.53 ± 1.15	4.5 ± 0.76	2.93 ± 0.64	1.09 ± 0.35	0.176 ± 0.003	0.963 ± 0.001
		IR	7.83 ± 1.73	5.94 ± 1.19	6.27 ± 1.41	6.11 ± 1.31	11.22 ± 2.24	7.84 ± 1.31	5.36 ± 1.35	2.03 ± 0.72	0.183 ± 0.004	0.978 ± 0.001
		DLIR	7.33 ± 1.71	5.64 ± 1.0	6.03 ± 1.25	5.83 ± 1.15	12.06 ± 2.35	8.24 ± 1.5	5.55 ± 1.22	2.07 ± 0.61	0.184 ± 0.004	0.979 ± 0.001
	AD	FBP	14.59 ± 2.68	10.3 ± 1.93	11.12 ± 2.22	10.71 ± 2.11	6.52 ± 1.21	4.78 ± 1.12	3.07 ± 0.69	1.13 ± 0.35	0.179 ± 0.004	0.962 ± 0.001

	IR	8.46 ± 1.98	6.2 ± 1.3	6.74 ± 1.59	6.47 ± 1.47	11.18 ± 2.38	8.32 ± 2.08	5.22 ± 1.3	1.96 ± 0.6	0.185 ± 0.004	0.977 ± 0.001
	DLIR	8.23 ± 1.75	5.69 ± 0.95	6.56 ± 1.4	6.12 ± 1.27	12.03 ± 2.29	8.51 ± 1.79	5.52 ± 1.23	2.05 ± 0.56	0.186 ± 0.004	0.978 ± 0.001
ADAC	FBP	15.14 ± 2.74	9.97 ± 1.83	10.17 ± 1.72	10.07 ± 1.78	6.9 ± 1.41	4.99 ± 1.08	2.92 ± 0.51	1.01 ± 0.31	0.177 ± 0.003	0.964 ± 0.001
	IR	8.99 ± 1.95	6.02 ± 1.23	6.12 ± 1.23	6.07 ± 1.23	11.79 ± 2.62	8.52 ± 1.99	4.93 ± 0.96	1.72 ± 0.48	0.183 ± 0.004	0.977 ± 0.001
	DLIR	8.71 ± 1.74	5.65 ± 1.0	6.09 ± 1.17	5.87 ± 1.11	12.25 ± 2.58	8.66 ± 1.93	4.98 ± 0.92	1.72 ± 0.44	0.185 ± 0.004	0.978 ± 0.001
ADAC2	FBP	12.95 ± 2.01	9.67 ± 1.8	11.22 ± 4.46	10.45 ± 3.48	6.91 ± 1.45	4.98 ± 1.2	3.53 ± 0.59	1.15 ± 0.31	0.18 ± 0.004	0.935 ± 0.005

	IR	7.31 ± 1.24	5.86 ± 1.15	7.31 ± 4.91	6.59 ± 3.63	11.69 ± 2.84	8.64 ± 2.2	6.26 ± 1.23	2.12 ± 0.53	0.188 ± 0.003	0.948 ± 0.005
	DLIR	7.28 ± 1.32	5.4 ± 0.86	7.38 ± 4.62	6.39 ± 3.46	12.39 ± 2.73	8.8 ± 2.05	6.35 ± 1.28	2.14 ± 0.48	0.189 ± 0.003	0.948 ± 0.005
AB	FBP	15.11 ± 2.35	14.13 ± 2.37	11.29 ± 2.06	12.71 ± 2.64	5.69 ± 1.03	4.5 ± 0.7	3.04 ± 0.78	1.19 ± 0.46	0.175 ± 0.003	0.957 ± 0.001
	IR	8.72 ± 1.59	8.51 ± 1.76	6.76 ± 1.43	7.64 ± 1.82	9.71 ± 1.96	7.83 ± 1.36	5.37 ± 1.98	2.14 ± 1.02	0.18 ± 0.003	0.974 ± 0.001
	DLIR	7.87 ± 1.37	7.41 ± 1.26	6.51 ± 1.35	6.96 ± 1.38	10.76 ± 1.89	8.21 ± 1.29	5.68 ± 1.74	2.2 ± 0.87	0.181 ± 0.003	0.975 ± 0.001
ABAC	FBP	14.49 ± 2.43	12.57 ± 2.24	11.57 ± 3.88	12.07 ± 3.2	5.84 ± 0.98	4.51 ± 0.93	2.89 ± 0.64	1.0 ± 0.36	0.177 ± 0.002	0.93 ± 0.005

		IR	8.17 ± 1.61	7.63 ± 1.45	7.49 ± 4.17	7.56 ± 3.12	9.95 ± 2.08	7.8 ± 1.58	5.08 ± 1.21	1.78 ± 0.64	0.185 ± 0.003	0.947 ± 0.005
		DLIR	7.61 ± 1.45	6.92 ± 1.09	7.3 ± 3.95	7.11 ± 2.9	10.88 ± 2.1	8.22 ± 1.63	5.31 ± 1.21	1.85 ± 0.62	0.186 ± 0.003	0.948 ± 0.005
	ABAC2	FBP	14.64 ± 2.33	12.24 ± 2.19	11.63 ± 4.14	11.93 ± 3.32	5.8 ± 1.19	4.55 ± 0.91	3.04 ± 0.71	1.12 ± 0.4	0.177 ± 0.002	0.931 ± 0.005
		IR	8.33 ± 1.59	7.5 ± 1.54	7.58 ± 4.68	7.54 ± 3.47	9.8 ± 2.34	7.94 ± 1.68	5.27 ± 1.29	1.97 ± 0.64	0.184 ± 0.003	0.947 ± 0.005
		DLIR	7.91 ± 1.48	6.62 ± 1.19	7.46 ± 4.39	7.04 ± 3.24	10.76 ± 2.24	8.27 ± 1.61	5.51 ± 1.31	2.0 ± 0.63	0.186 ± 0.003	0.947 ± 0.005
11	AU	FBP	17.94 ± 2.54	12.88 ± 2.21	12.92 ± 2.21	12.9 ± 2.2	5.28 ± 0.76	3.87 ± 0.72	2.27 ± 0.55	0.86 ± 0.36	0.175 ± 0.003	0.951 ± 0.002

	IR	10.01 ± 1.65	7.67 ± 1.5	7.64 ± 1.48	7.66 ± 1.49	9.12 ± 1.51	6.8 ± 1.3	3.98 ± 0.99	1.48 ± 0.56	0.181 ± 0.004	0.974 ± 0.001
	DLIR	9.11 ± 1.45	6.56 ± 1.11	7.02 ± 1.37	6.79 ± 1.27	10.42 ± 1.49	7.51 ± 1.43	4.28 ± 1.04	1.54 ± 0.61	0.183 ± 0.004	0.976 ± 0.001
AD	FBP	17.25 ± 2.76	13.72 ± 2.68	13.49 ± 2.69	13.6 ± 2.68	5.23 ± 0.79	3.67 ± 0.57	2.59 ± 0.59	1.0 ± 0.31	0.176 ± 0.003	0.948 ± 0.002
	IR	9.82 ± 1.89	8.34 ± 1.85	7.97 ± 1.73	8.15 ± 1.8	8.92 ± 1.57	6.27 ± 1.12	4.44 ± 0.91	1.73 ± 0.5	0.182 ± 0.003	0.971 ± 0.001
	DLIR	9.26 ± 1.75	7.06 ± 1.25	7.42 ± 1.51	7.24 ± 1.39	10.14 ± 1.67	6.95 ± 1.15	4.57 ± 0.9	1.72 ± 0.48	0.183 ± 0.004	0.974 ± 0.001
ADAC	FBP	18.33 ± 3.23	12.66 ± 2.66	13.0 ± 2.23	12.83 ± 2.45	5.6 ± 1.07	3.76 ± 0.66	2.69 ± 0.41	1.0 ± 0.28	0.178 ± 0.002	0.951 ± 0.002

	IR	10.73 ± 2.16	7.71 ± 1.71	7.73 ± 1.57	7.72 ± 1.64	9.6 ± 2.04	6.48 ± 1.26	4.62 ± 0.92	1.73 ± 0.52	0.182 ± 0.003	0.972 ± 0.001
	DLIR	10.1 ± 1.97	6.82 ± 1.32	7.22 ± 1.33	7.02 ± 1.33	10.62 ± 2.07	6.92 ± 1.29	4.86 ± 0.89	1.82 ± 0.47	0.182 ± 0.004	0.974 ± 0.001
ADAC2	FBP	16.97 ± 3.47	12.67 ± 2.52	13.77 ± 4.37	13.22 ± 3.6	5.25 ± 1.12	3.81 ± 0.74	2.24 ± 0.37	0.72 ± 0.28	0.177 ± 0.003	0.923 ± 0.005
	IR	9.7 ± 2.31	7.54 ± 1.58	8.92 ± 4.85	8.23 ± 3.66	9.01 ± 2.16	6.59 ± 1.48	3.89 ± 0.51	1.28 ± 0.36	0.185 ± 0.004	0.944 ± 0.005
	DLIR	9.1 ± 2.12	6.49 ± 1.09	8.53 ± 4.54	7.51 ± 3.45	10.29 ± 2.18	7.04 ± 1.52	4.21 ± 0.58	1.39 ± 0.36	0.187 ± 0.003	0.945 ± 0.005
AB	FBP	18.23 ± 3.03	16.55 ± 2.54	13.71 ± 2.37	15.13 ± 2.83	4.83 ± 0.81	3.43 ± 0.65	2.2 ± 0.64	0.69 ± 0.28	0.175 ± 0.003	0.945 ± 0.001

	IR	10.48 ± 1.93	9.93 ± 1.82	8.2 ± 1.69	9.07 ± 1.96	8.29 ± 1.68	5.97 ± 1.23	3.83 ± 1.32	1.23 ± 0.47	0.18 ± 0.002	0.969 ± 0.001
	DLIR	9.5 ± 1.71	8.66 ± 1.29	7.56 ± 1.57	8.11 ± 1.54	9.53 ± 1.87	6.61 ± 1.35	4.23 ± 1.27	1.36 ± 0.47	0.179 ± 0.003	0.972 ± 0.001
ABAC	FBP	18.58 ± 3.03	14.76 ± 2.68	13.9 ± 3.82	14.33 ± 3.32	4.81 ± 0.75	3.51 ± 0.63	2.18 ± 0.55	0.72 ± 0.33	0.175 ± 0.002	0.918 ± 0.005
	IR	10.7 ± 2.24	8.91 ± 1.83	8.78 ± 4.14	8.85 ± 3.19	8.22 ± 1.52	6.13 ± 1.23	3.81 ± 1.04	1.28 ± 0.54	0.183 ± 0.003	0.942 ± 0.005
	DLIR	9.55 ± 1.81	7.94 ± 1.55	8.22 ± 3.78	8.08 ± 2.89	9.35 ± 1.62	6.94 ± 1.32	4.16 ± 1.08	1.36 ± 0.56	0.184 ± 0.003	0.944 ± 0.005
ABAC2	FBP	18.37 ± 2.99	14.73 ± 2.19	13.84 ± 3.73	14.29 ± 3.08	4.8 ± 0.89	3.73 ± 0.68	2.21 ± 0.34	0.76 ± 0.22	0.176 ± 0.002	0.919 ± 0.005

		IR	10.59 ± 2.13	8.92 ± 1.5	8.79 ± 4.14	8.85 ± 3.11	8.19 ± 1.69	6.63 ± 1.33	3.84 ± 0.61	1.34 ± 0.32	0.182 ± 0.003	0.942 ± 0.004
		DLIR	9.47 ± 1.9	7.68 ± 1.08	8.3 ± 4.0	7.99 ± 2.94	9.42 ± 1.75	7.31 ± 1.46	4.21 ± 0.71	1.46 ± 0.33	0.183 ± 0.003	0.944 ± 0.005
13	AU	FBP	21.03 ± 3.45	15.35 ± 2.8	15.41 ± 3.21	15.38 ± 3.0	4.56 ± 0.84	3.07 ± 0.48	1.91 ± 0.39	0.67 ± 0.3	0.174 ± 0.003	0.938 ± 0.002
		IR	12.34 ± 2.46	9.13 ± 1.95	9.11 ± 2.23	9.12 ± 2.09	7.9 ± 1.61	5.27 ± 0.88	3.18 ± 0.67	1.12 ± 0.44	0.182 ± 0.003	0.969 ± 0.001
		DLIR	11.33 ± 2.18	7.69 ± 1.39	8.3 ± 1.64	8.0 ± 1.55	9.08 ± 1.68	5.93 ± 0.94	3.52 ± 0.63	1.23 ± 0.41	0.183 ± 0.003	0.972 ± 0.001
	AD	FBP	21.47 ± 2.57	16.53 ± 2.99	16.89 ± 3.35	16.71 ± 3.17	4.45 ± 0.77	3.23 ± 0.62	1.97 ± 0.39	0.75 ± 0.28	0.176 ± 0.002	0.934 ± 0.002

	IR	12.81 ± 1.69	9.81 ± 2.0	10.2 ± 2.57	10.0 ± 2.3	7.57 ± 1.36	5.55 ± 1.2	3.3 ± 0.63	1.28 ± 0.37	0.181 ± 0.004	0.966 ± 0.001
	DLIR	11.79 ± 1.58	8.5 ± 1.5	9.3 ± 2.08	8.9 ± 1.85	8.65 ± 1.4	6.16 ± 1.24	3.6 ± 0.7	1.33 ± 0.38	0.181 ± 0.004	0.969 ± 0.001
ADAC	FBP	21.61 ± 3.38	15.41 ± 2.63	15.38 ± 3.14	15.4 ± 2.89	4.43 ± 0.77	3.09 ± 0.65	1.89 ± 0.38	0.61 ± 0.28	0.175 ± 0.002	0.937 ± 0.002
	IR	12.96 ± 2.56	9.13 ± 1.75	9.26 ± 2.07	9.2 ± 1.91	7.48 ± 1.35	5.22 ± 1.21	3.15 ± 0.66	1.02 ± 0.44	0.182 ± 0.003	0.967 ± 0.001
	DLIR	11.46 ± 1.95	7.82 ± 1.33	8.56 ± 1.92	8.19 ± 1.69	8.68 ± 1.57	5.85 ± 1.21	3.61 ± 0.77	1.13 ± 0.45	0.181 ± 0.003	0.97 ± 0.001
ADAC2	FBP	20.54 ± 3.24	15.46 ± 2.63	16.13 ± 3.88	15.79 ± 3.32	4.44 ± 0.86	3.13 ± 0.55	2.11 ± 0.47	0.76 ± 0.34	0.176 ± 0.003	0.91 ± 0.005

	IR	12.28 ± 2.19	9.23 ± 1.66	10.24 ± 4.35	9.73 ± 3.32	7.56 ± 1.63	5.48 ± 1.11	3.66 ± 0.84	1.34 ± 0.54	0.183 ± 0.003	0.939 ± 0.005
	DLIR	11.06 ± 2.02	7.96 ± 1.19	9.55 ± 4.17	8.76 ± 3.16	8.76 ± 1.78	6.02 ± 1.2	3.81 ± 0.78	1.39 ± 0.5	0.184 ± 0.003	0.941 ± 0.005
AB	FBP	21.7 ± 3.56	18.94 ± 3.48	15.49 ± 2.82	17.22 ± 3.59	4.01 ± 0.81	3.07 ± 0.5	2.1 ± 0.45	0.76 ± 0.34	0.175 ± 0.003	0.934 ± 0.002
	IR	12.78 ± 2.55	11.51 ± 2.47	9.3 ± 1.94	10.41 ± 2.48	6.72 ± 1.49	5.31 ± 0.97	3.63 ± 0.78	1.33 ± 0.49	0.181 ± 0.003	0.965 ± 0.001
	DLIR	11.74 ± 2.1	10.02 ± 2.05	8.44 ± 1.84	9.23 ± 2.1	7.75 ± 1.47	6.04 ± 1.06	3.96 ± 0.86	1.43 ± 0.52	0.179 ± 0.003	0.968 ± 0.001
ABAC	FBP	21.92 ± 3.7	17.23 ± 2.81	15.71 ± 3.74	16.47 ± 3.39	4.42 ± 0.74	3.23 ± 0.52	1.91 ± 0.42	0.69 ± 0.38	0.175 ± 0.002	0.907 ± 0.005

	IR	13.04 ± 2.67	10.4 ± 1.98	9.83 ± 4.05	10.12 ± 3.19	7.47 ± 1.47	5.65 ± 0.97	3.28 ± 0.72	1.21 ± 0.57	0.183 ± 0.003	0.938 ± 0.005
	DLIR	11.77 ± 2.12	9.1 ± 1.56	9.09 ± 3.73	9.1 ± 2.85	8.65 ± 1.52	6.32 ± 1.2	3.5 ± 0.64	1.23 ± 0.51	0.183 ± 0.003	0.941 ± 0.005
ABAC2	FBP	20.99 ± 4.19	16.93 ± 3.14	15.98 ± 3.54	16.46 ± 3.37	4.22 ± 0.71	3.33 ± 0.73	1.98 ± 0.46	0.76 ± 0.35	0.175 ± 0.002	0.908 ± 0.005
	IR	12.27 ± 2.92	10.28 ± 2.21	9.98 ± 3.91	10.13 ± 3.17	7.17 ± 1.4	5.75 ± 1.37	3.47 ± 0.8	1.31 ± 0.54	0.182 ± 0.002	0.938 ± 0.005
	DLIR	11.2 ± 2.35	8.81 ± 1.72	9.3 ± 3.73	9.06 ± 2.91	8.24 ± 1.42	6.43 ± 1.37	3.7 ± 0.75	1.36 ± 0.48	0.182 ± 0.003	0.941 ± 0.005

Data are presented as mean ± standard deviation. AB, arms placed atop the belly; ABAC, arms placed atop the belly with a single layer of air cushions; ABAC2, arms placed atop the belly with double-layered air cushions; AD, arms down alongside the torso; ADAC, arms down alongside the torso with a single layer of air cushions; ADAC2, arms down alongside the torso with double-layered air cushions; AU, arms up; CNR, contrast-to-noise ratio; DLIR, deep learning image reconstruction; FBP, filtered back projection; IR, iterative reconstruction; SNR, signal-to-noise ratio; SSIM, structural similarity index.

Supplementary Table S3. Qualitative analysis of CT images according to noise index, arm position, and reconstruction algorithm

Noise index	Reconstruction	Arm position	Sharpness	Noise	Artifacts	Overall Image Quality
Reference of standard	FBP	AU	3	3	4	3
9	FBP	AU	2.2 ± 0.3	2.0 ± 0.0	4.0 ± 0.0	2.1 ± 0.2
		AD	2.0 ± 0.0	1.1 ± 0.2	1.1 ± 0.2	1.1 ± 0.2
		ADAC	2.0 ± 0.0	1.0 ± 0.0	2.0 ± 0.0	1.8 ± 0.3
		ADAC2	2.4 ± 0.2	1.0 ± 0.0	2.0 ± 0.0	2.0 ± 0.0
		AB	1.2 ± 0.3	1.0 ± 0.0	1.0 ± 0.0	1.0 ± 0.0
		ABAC	1.8 ± 0.3	1.3 ± 0.3	1.2 ± 0.3	1.4 ± 0.2
		ABAC2	1.9 ± 0.2	1.4 ± 0.2	2.0 ± 0.0	2.0 ± 0.0
	IR	AU	2.2 ± 0.3	3.0 ± 0.0	4.0 ± 0.0	2.0 ± 0.0
		AD	1.9 ± 0.2	1.4 ± 0.2	2.0 ± 0.0	2.1 ± 0.2
		ADAC	1.9 ± 0.2	1.6 ± 0.2	2.0 ± 0.0	2.0 ± 0.0
		ADAC2	2.0 ± 0.0	1.3 ± 0.3	2.1 ± 0.2	2.3 ± 0.3
		AB	1.9 ± 0.2	1.5 ± 0.0	1.5 ± 0.0	1.5 ± 0.0
		ABAC	2.0 ± 0.0	2.0 ± 0.0	2.0 ± 0.0	2.1 ± 0.2
		ABAC2	2.1 ± 0.2	2.2 ± 0.3	2.4 ± 0.2	2.1 ± 0.2

	DLIR	AU	2.3 ± 0.3	3.0 ± 0.0	4.0 ± 0.0	2.5 ± 0.0
		AD	1.2 ± 0.3	1.9 ± 0.2	2.0 ± 0.0	2.0 ± 0.0
		ADAC	1.6 ± 0.2	2.0 ± 0.0	2.0 ± 0.0	2.8 ± 0.3
		ADAC2	1.7 ± 0.3	2.7 ± 0.3	2.2 ± 0.3	2.4 ± 0.2
		AB	1.0 ± 0.0	2.5 ± 0.0	2.0 ± 0.0	2.0 ± 0.0
		ABAC	2.0 ± 0.0	2.4 ± 0.2	2.4 ± 0.2	2.0 ± 0.0
		ABAC2	2.3 ± 0.3	2.5 ± 0.0	3.0 ± 0.0	2.5 ± 0.0
11	FBP	AU	2.4 ± 0.4	2.0 ± 0.0	4.0 ± 0.0	2.0 ± 0.0
		AD	2.0 ± 0.0	1.5 ± 0.4	1.8 ± 0.3	2.0 ± 0.0
		ADAC	3.0 ± 0.0	1.8 ± 0.3	2.1 ± 0.2	2.0 ± 0.0
		ADAC2	3.0 ± 0.0	1.4 ± 0.2	2.5 ± 0.0	2.2 ± 0.3
		AB	2.0 ± 0.0	2.0 ± 0.0	1.8 ± 0.3	1.5 ± 0.0
		ABAC	2.1 ± 0.2	1.5 ± 0.0	1.5 ± 0.0	2.0 ± 0.0
		ABAC2	2.7 ± 0.3	2.0 ± 0.0	2.0 ± 0.0	2.0 ± 0.0
	IR	AU	2.1 ± 0.2	2.7 ± 0.3	4.0 ± 0.0	2.8 ± 0.3
		AD	2.0 ± 0.0	1.9 ± 0.2	2.1 ± 0.2	2.3 ± 0.3
		ADAC	2.8 ± 0.3	2.2 ± 0.4	2.3 ± 0.3	2.2 ± 0.3
		ADAC2	2.9 ± 0.2	2.3 ± 0.3	2.3 ± 0.4	2.7 ± 0.3
		AB	2.5 ± 0.0	2.5 ± 0.0	2.0 ± 0.0	2.0 ± 0.0
		ABAC	2.7 ± 0.3	2.2 ± 0.3	2.1 ± 0.2	2.3 ± 0.3
		ABAC2	2.8 ± 0.3	2.4 ± 0.2	2.3 ± 0.3	2.3 ± 0.3

	DLIR	AU	2.4 ± 0.2	3.0 ± 0.0	4.0 ± 0.0	3.0 ± 0.0
		AD	2.0 ± 0.0	2.0 ± 0.0	2.5 ± 0.4	2.8 ± 0.3
		ADAC	2.6 ± 0.2	2.5 ± 0.0	2.6 ± 0.2	2.8 ± 0.3
		ADAC2	2.8 ± 0.3	2.9 ± 0.2	2.8 ± 0.3	3.0 ± 0.0
		AB	2.0 ± 0.0	2.5 ± 0.0	3.0 ± 0.0	2.0 ± 0.0
		ABAC	2.3 ± 0.3	3.0 ± 0.0	3.0 ± 0.0	2.2 ± 0.3
		ABAC2	2.5 ± 0.0	3.0 ± 0.0	3.0 ± 0.0	3.0 ± 0.0
13	FBP	AU	3.9 ± 0.2	2.0 ± 0.0	4.0 ± 0.0	2.1 ± 0.2
		AD	3.0 ± 0.0	2.0 ± 0.0	2.0 ± 0.0	2.0 ± 0.0
		ADAC	3.0 ± 0.0	2.0 ± 0.0	2.3 ± 0.3	2.2 ± 0.3
		ADAC2	3.0 ± 0.0	2.0 ± 0.0	3.0 ± 0.0	3.0 ± 0.0
		AB	2.6 ± 0.2	2.0 ± 0.0	2.0 ± 0.0	1.5 ± 0.0
		ABAC	2.9 ± 0.2	2.0 ± 0.0	2.0 ± 0.0	2.0 ± 0.0
		ABAC2	3.0 ± 0.0	2.0 ± 0.0	3.0 ± 0.0	2.8 ± 0.3
	IR	AU	2.7 ± 0.3	3.0 ± 0.0	4.0 ± 0.0	2.8 ± 0.3
		AD	2.9 ± 0.2	2.7 ± 0.3	2.6 ± 0.2	2.7 ± 0.3
		ADAC	3.0 ± 0.0	3.0 ± 0.0	2.5 ± 0.0	3.0 ± 0.0
		ADAC2	3.0 ± 0.0	2.8 ± 0.3	2.6 ± 0.2	3.0 ± 0.0
		AB	3.0 ± 0.0	2.5 ± 0.0	2.0 ± 0.0	2.0 ± 0.0
		ABAC	3.0 ± 0.0	3.0 ± 0.0	2.6 ± 0.2	3.0 ± 0.0
		ABAC2	3.0 ± 0.0	3.0 ± 0.0	3.3 ± 0.3	3.0 ± 0.0

DLIR	AU	2.9 ± 0.7	3.5 ± 0.0	4.0 ± 0.0	3.2 ± 0.3
	AD	2.8 ± 0.3	2.8 ± 0.3	2.8 ± 0.3	3.0 ± 0.0
	ADAC	3.0 ± 0.0	3.0 ± 0.0	3.0 ± 0.0	3.0 ± 0.0
	ADAC2	3.0 ± 0.0	3.0 ± 0.0	3.7 ± 0.3	3.0 ± 0.0
	AB	2.2 ± 0.3	3.0 ± 0.0	3.0 ± 0.0	3.0 ± 0.0
	ABAC	3.0 ± 0.0	3.0 ± 0.0	3.0 ± 0.0	3.0 ± 0.0
	ABAC2	3.0 ± 0.0	3.0 ± 0.0	4.0 ± 0.0	3.0 ± 0.0

Data are presented as mean ± standard deviation unless otherwise specified.

Interobserver agreement for the subjective image quality evaluation was high (intraclass correlation coefficient, 0.839; 95% confidence interval, 0.818–0.857; $p < 0.001$).

AB, arms placed atop the belly; ABAC, arms placed atop the belly with a single layer air cushions; ABAC2, arms placed atop the belly with double-layered air cushions; AD, arms down alongside the torso; ADAC, arms down alongside the torso with a single layer air cushions; ADAC2, arms down alongside the torso with double-layered air cushions; AU, arms up; DLIR, deep learning image reconstruction; FBP, filtered back projection; IR, iterative reconstruction.

Supplementary Figure legends

Supplementary Figure S1.

Representative axial abdominal CT image illustrating the placement of circular regions of interest (ROIs) used for quantitative image analysis. Circular ROIs with a uniform area of approximately 50 mm² were placed at predefined locations. For image noise measurements, central ROIs were positioned in the fat immediately anterior to the aorta (blue circle), whereas peripheral ROIs were placed within the subcutaneous fat layer of the anterior and bilateral abdominal walls (blue circles). For signal-to-noise ratio (SNR) and contrast-to-noise ratio (CNR) measurements, additional ROIs were drawn within the liver parenchyma (purple circle) and within the aortic lumen (red circle), with the latter serving as the reference structure for CNR calculation. All ROIs were subsequently copied and pasted across all image series to ensure identical anatomical level, location, and size for quantitative analysis.

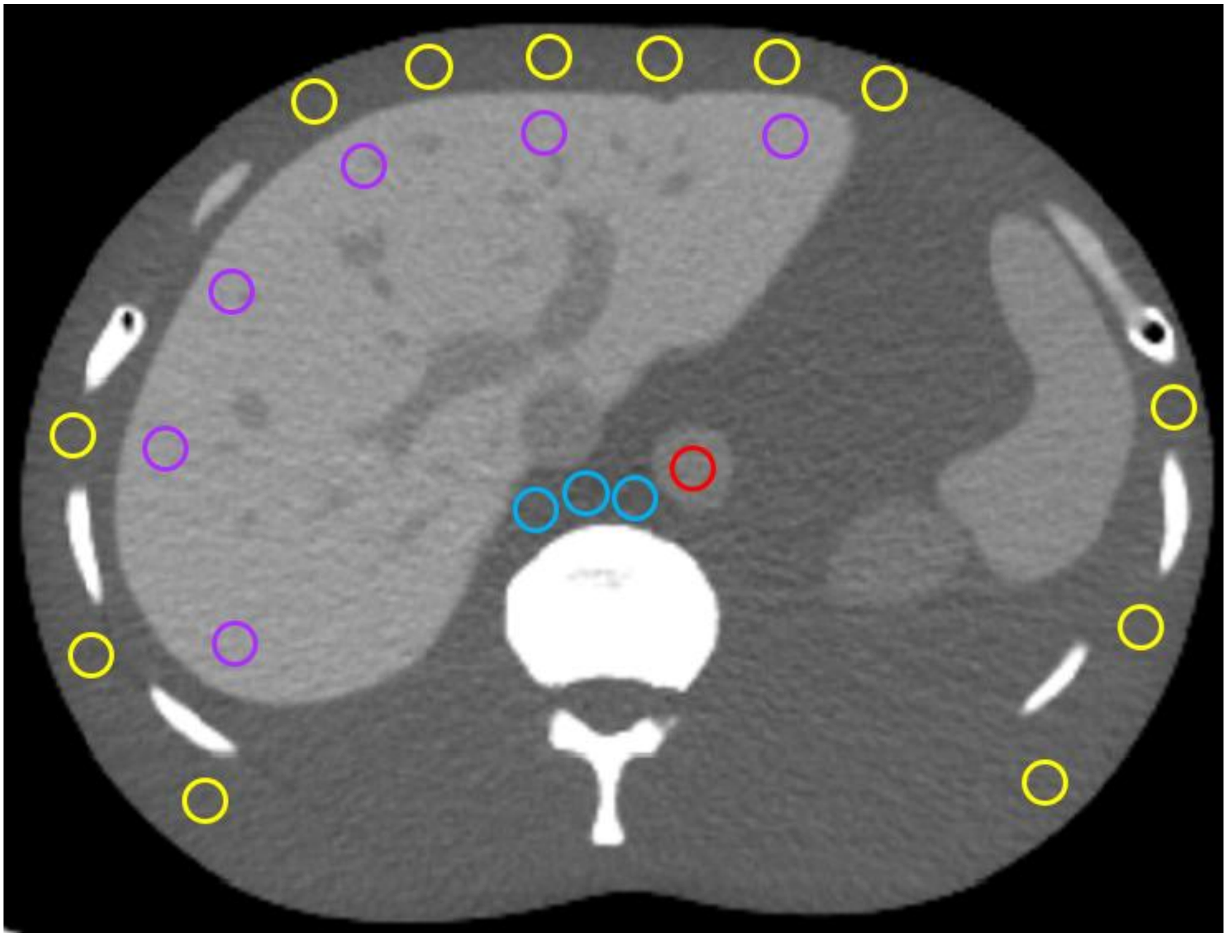

Supplementary Figure S2.

Representative axial abdominal CT image illustrating the application of a predefined mask used to define a specific region of interest (ROI) for quantitative analysis of SSIM and blur metrics. The mask was applied to exclude areas potentially affected by arm positioning, thereby enabling consistent and reproducible assessment of image quality variations attributable to arm interference across different scanning conditions.

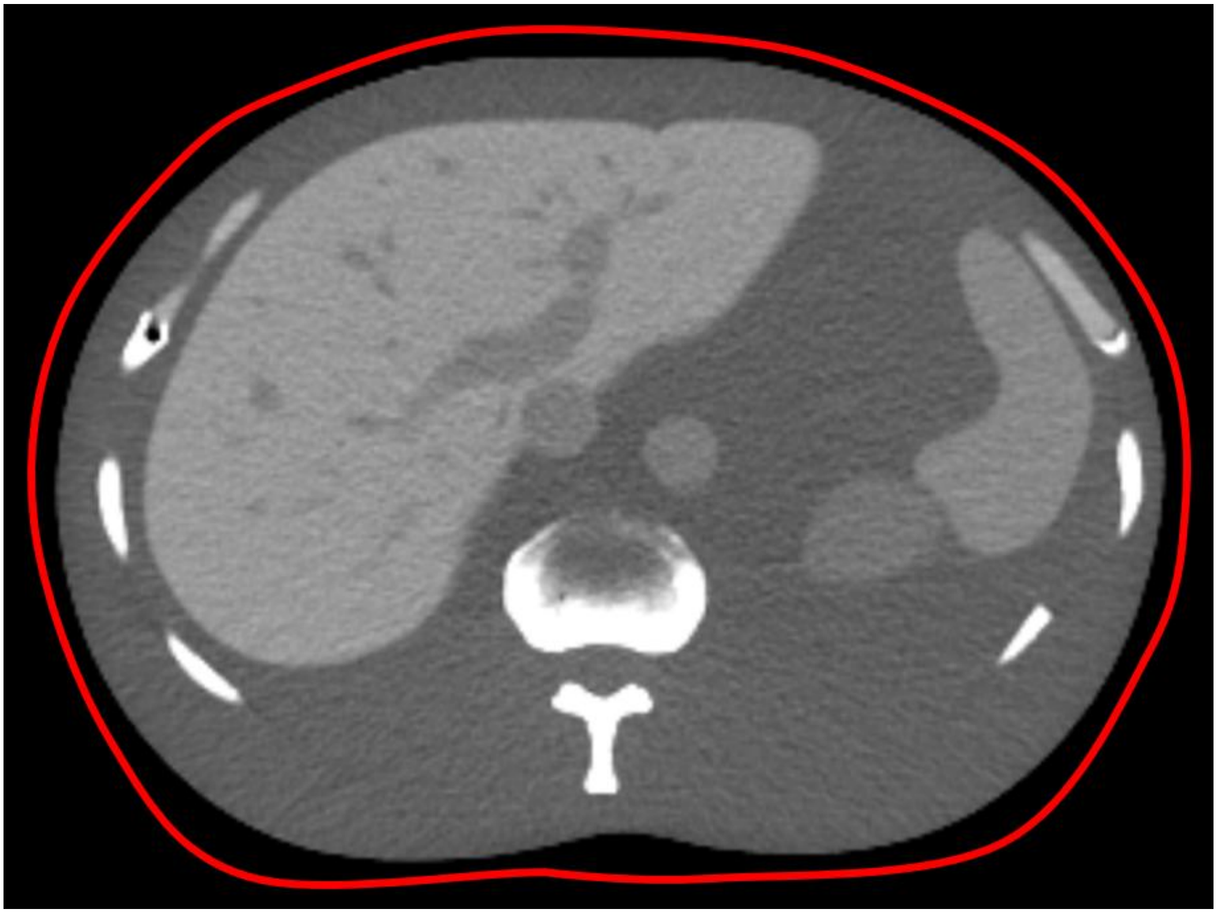

Supplementary Figure S3.

Quantitative image quality metrics stratified by noise index settings (NI = 9, 11, and 13). Panels (a–f), (g–l), and (m–r) correspond to NI values of 9, 11, and 13, respectively. For each noise index, image noise (central and peripheral), liver signal-to-noise ratio (SNR), liver contrast-to-noise ratio (CNR), blur metric, and structural similarity index (SSIM) are shown according to arm position and reconstruction method. The overall patterns of variation across arm positions and reconstruction techniques were consistent across different noise index settings. AB, arms placed atop the belly; ABAC, arms placed atop the belly with a single layer of air cushion; ABAC2, arms placed atop the belly with double-layered air cushions; AD, arms down alongside the torso; ADAC, arms down alongside the torso with a single layer of air cushion; ADAC2, arms down alongside the torso with double-layered air cushions; AU, arms up; CNR, contrast-to-noise ratio; DLIR, deep learning image reconstruction; FBP, filtered back projection; IR, iterative reconstruction; SNR, signal-to-noise ratio; SSIM, structural similarity index.

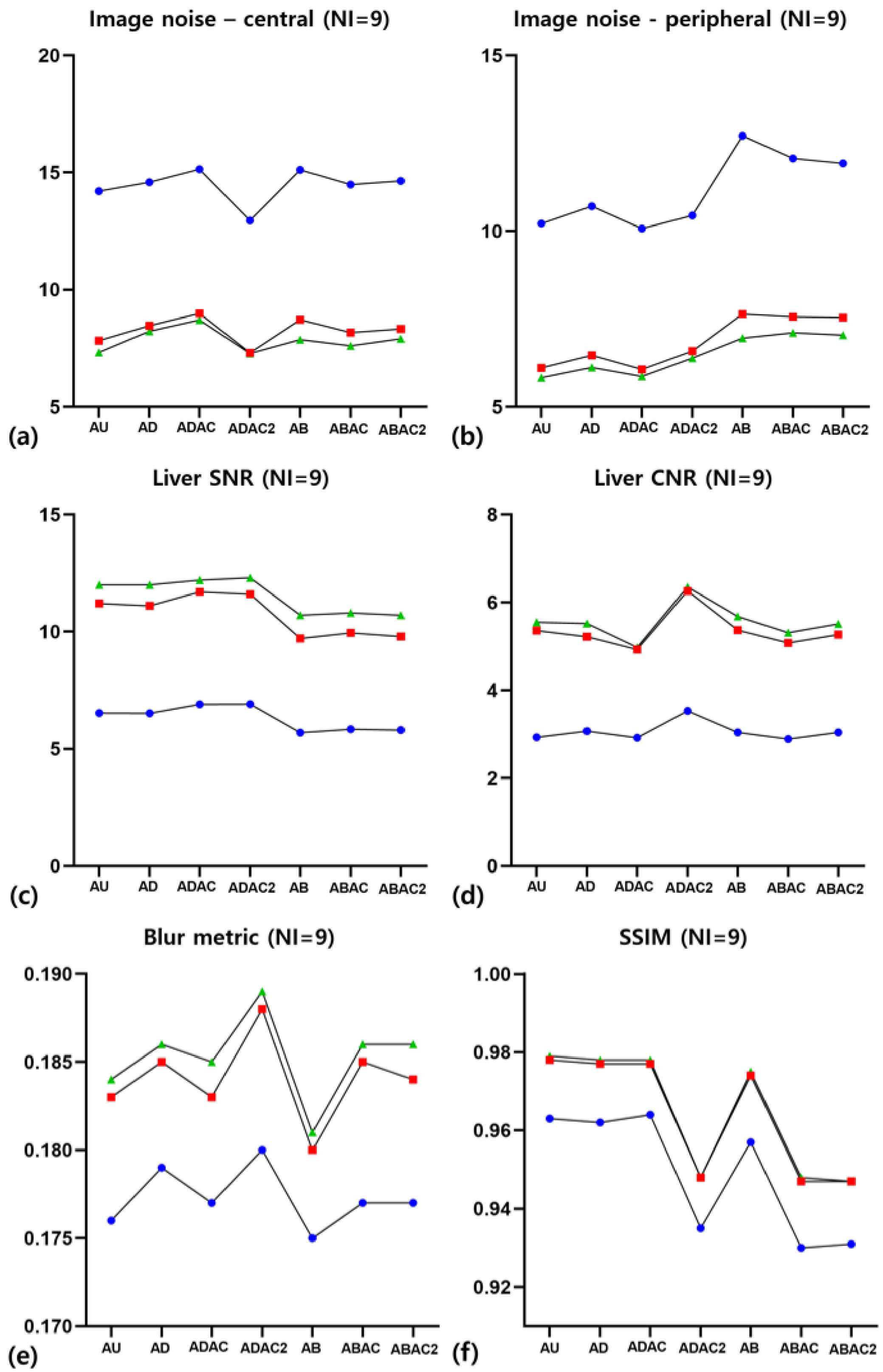
Image noise – central (NI=9)
Image noise - peripheral (NI=9)
Liver SNR (NI=9)
Liver CNR (NI=9)
Blur metric (NI=9)
SSIM (NI=9)
AU
AD
ADAC
ADAC2
AB
ABAC
ABAC2
(a)
(b)
(c)
(d)
(e)
(f)

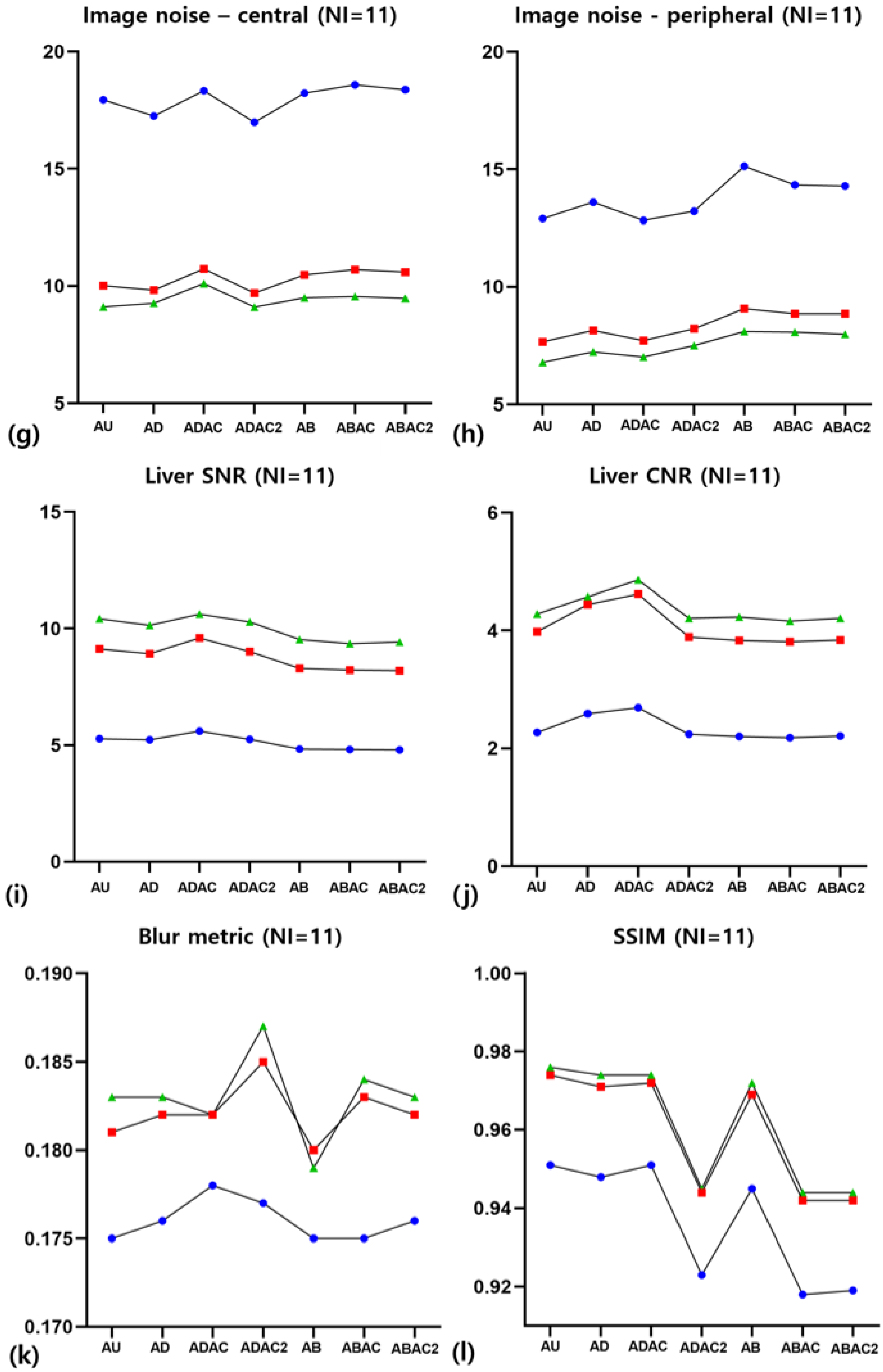
Image noise – central (NI=11)
20
15
10
5
AU
AD
ADAC
ADAC2
AB
ABAC
ABAC2
(g)
Image noise - peripheral (NI=11)
20
15
10
5
AU
AD
ADAC
ADAC2
AB
ABAC
ABAC2
(h)
Liver SNR (NI=11)
15
10
5
0
AU
AD
ADAC
ADAC2
AB
ABAC
ABAC2
(i)
Liver CNR (NI=11)
6
4
2
0
AU
AD
ADAC
ADAC2
AB
ABAC
ABAC2
(j)
Blur metric (NI=11)
0.190
0.185
0.180
0.175
0.170
AU
AD
ADAC
ADAC2
AB
ABAC
ABAC2
(k)
SSIM (NI=11)
1.00
0.98
0.96
0.94
0.92
AU
AD
ADAC
ADAC2
AB
ABAC
ABAC2
(l)

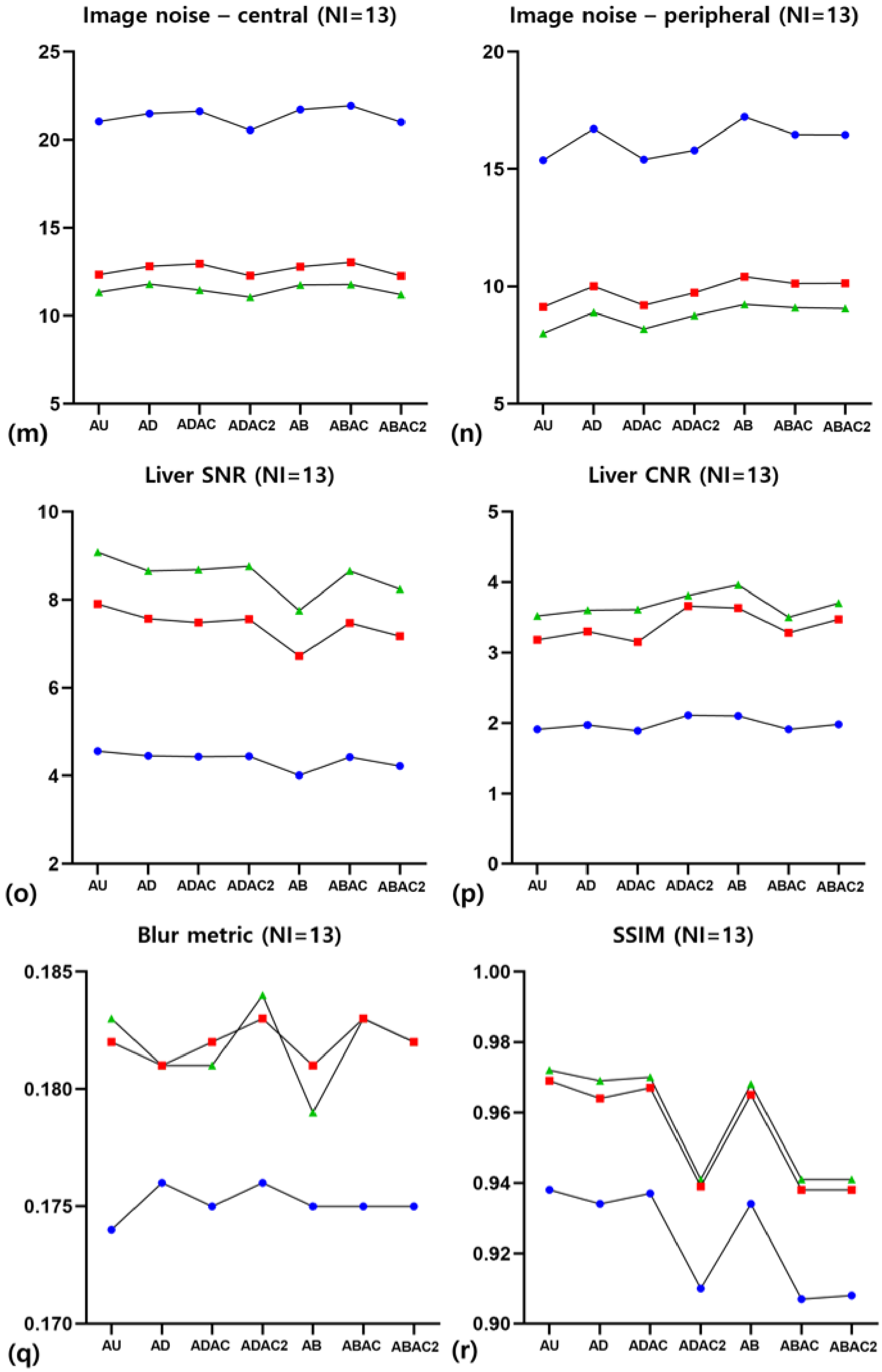
Image noise – central (NI=13)
(m)
Image noise – peripheral (NI=13)
(n)
Liver SNR (NI=13)
(o)
Liver CNR (NI=13)
(p)
Blur metric (NI=13)
(q)
SSIM (NI=13)
(r)
AU
AD
ADAC
ADAC2
AB
ABAC
ABAC2

Supplementary Figure S4.

Representative axial abdominal CT images demonstrating qualitative differences in image quality according to arm position and reconstruction method. The images illustrate variations in sharpness, image noise, artifact severity, and overall diagnostic image quality across different arm configurations and reconstruction algorithms, complementing the qualitative comparisons shown in Figure 4. Columns are arranged according to arm position in the following order: arms-up (AU), arms-down alongside the torso (AD), arms-down with a single air cushion (ADAC), arms-down with double air cushions (ADAC2), arms-on-belly (AB), arms-on-belly with a single air cushion (ABAC), and arms-on-belly with double air cushions (ABAC2). Image panels a–g correspond to filtered back projection (FBP), panels h–n to iterative reconstruction (IR), and panels o–u to deep learning–based image reconstruction (DLIR).

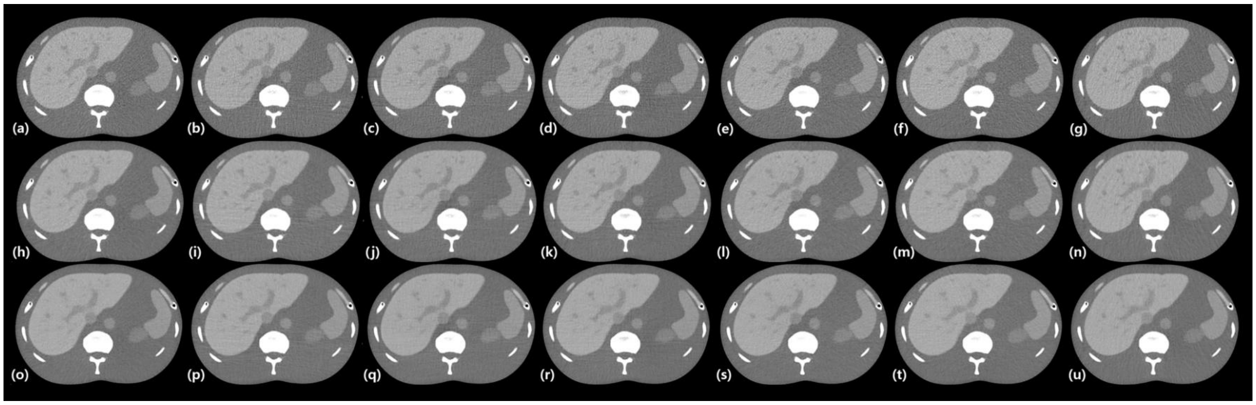

Supplement: Supplementary file 1 — Additional file 1: Table S1. Radiation dose according to different arm positions during abdominal CT scans. Table S2. Comprehensive quantitative analysis results. Table S3. Qualitative analysis of CT images according to noise index, arm position, and reconstruction algorithm. Figure S1. Representative axial abdominal CT image illustrating the placement of circular regions of interest (ROIs) used for quantitative image analysis. Figure S2. Representative axial abdominal CT image illustrating the application of a predefined mask used to define a specific region of interest (ROI) for quantitative analysis of SSIM and blur metrics. Figure S3. Quantitative image quality metrics stratified by noise index settings (NI = 9, 11, and 13). Panels (a–f), (g–l), and (m–r) correspond to NI values of 9, 11, and 13, respectively. Figure S4. Representative axial abdominal CT images demonstrating qualitative differences in image quality according to arm position and reconstruction method. [file 41747_2026_722_MOESM1_ESM.pdf]
